# Efficient biotechnological production of *N*-(Hydroxycinnamoyl)tyramines by utilizing plant-derived enzymes in a bench-scale bioreactor with in situ product precipitation

**DOI:** 10.1007/s00253-026-13997-6

**Published:** 2026-08-26

**Authors:** Israa Abuobida Mohamed, Qais Al-Khawlani, Raika Milde, Niels Valentin Heise, Martin Dippe, Mohamed Nagia, Ludger A. Wessjohann, Markus Pietzsch, Bodo Moritz

**Affiliations:** 1 Institute of Pharmacy, Martin-Luther-University Halle-Wittenberg, Weinbergweg 22, 06120 Halle (Saale), Germany; 2 Leibniz-Institute of Plant Biochemistry, Department of Bioorganic Chemistry, Weinberg 3, 06120 Halle, Germany; 3 Institute of Chemistry, Martin-Luther-University Halle-Wittenberg, Kurt-Mothes-Straße 2, 06120 Halle, Germany; 4 Department of Chemistry of Natural Compounds, Pharmaceutical and Drug Industries Research Institute, National Research Center, El Buhouth St. 33, 12622 Cairo, Egypt

**Keywords:** Hydroxycinnamic acid amide, Phenyl propanoic acids, Feruloyltyramine, Coumaroyltyramine, High cell density fermentation, Metabolic engineering, Product recovery

## Abstract

**Abstract:**

The efficient recovery of plant polyphenolic bioactives is facing technological challenges of low concentrations and complex mixtures starting from plant tissue. In the search of alternatives, we developed a high-yield biotechnological production process for hydroxycinnamic acid–tyramine conjugates in *Escherichia coli* by converting soluble substrates into insoluble products. A recombinant plant-enzyme cascade was established by co-expression of *Arabidopsis thaliana* 4-coumarate:CoA ligase and *Solanum tuberosum* hydroxycinnamoyl-CoA:tyramine *N*-(hydroxycinnamoyl)transferase. We overcame initial enzymatic limitations by developing a temperature adapted and sequentially phased bioprocess on a 1 L reactor scale. With 5 mM/h substrate supply rate we achieved 31.9 g/L (102 mM) of feruloyltyramine (FerT), 14.4 g/L (50.7 mM) coumaroyltyramine, and 14.6 g/L (46.5 mM) isoferuloyltyramine with a space time yield of 1.75 g *L^−1^ *h^−1^ FerT. The vast majority of the product was obtained as filterable precipitate, simplifying the downstream process compared to classical procedures. Washed filter cake from a FerT production after filtration via 16–40 µm glass filter, showed 99% mass content of FerT. Product identity was confirmed by NMR after recrystallization. The newly developed process is robust, cost-effective and scalable, paving the way for commercial applications of biotechnologically produced hydroxycinnamic acid–tyramines.

**Key points:**

• *Plant derived enzymes expressed at low temperature enable high productivity*

• *In situ precipitated product is readily filtered from fermentation broth*

• *Carbon transfer rate mirrors toxicity of phenolics and guide process optimization*

**Supplementary Information:**

The online version contains supplementary material available at https://doi.org/10.1007/s00253-026-13997-6.

## Introduction

Hydroxycinnamic acids (HCAs) such as coumaric acid (CouA), ferulic acid (FerA) and caffeic acid (CafA) are synthesized in all plants mainly from phenylalanine and are the precursors of monolignols, flavonoids, coumarins, stilbenes and many more (Marchiosi et al. 2020; Vogt 2010; Zhu et al. 2024). The major mass fraction of synthesized HCAs is activated by 4-coumarate-CoA ligase (4CL) to CoA-thioester and finally reduced to monolignols for the synthesis of lignin. A minor fraction of the HCA CoA-thioesters is transferred to phenolic amines by hydroxycinnamoyl-CoA:tyramine N-(hydroxycinnamoyl)transferase (THT) to form hydroxycinnamic acid amides (HCAAs). Phenolic amines like tyramine (TyN) and tryptamine have been shown to be synthesized preferentially upon heat and biotic stress (Lehmann and Pollmann 2009). The amides of HCAs and phenolic amines have been shown to be involved in plant defense against pathogens acting as phytoalexins (Zeiss et al. 2021). An increase in feruloyltyramine (FerT) synthesis was reported in several studies, e.g., (1) in tobacco leaves after infection with tobacco mosaic virus (Negrel and Jeandet 1987), (2) in potato leaves after infection with the oomycete *Phytophthora infestans*, the causative agent of the devastating late blight disease (Keller et al. 1996; Schmidt et al. 1999; Zacarés et al. 2007), and (3) in *Arabidopsis thaliana* leaves after infection with *Pseudomonas syringae* (Macoy et al. 2022). The concentration of FerT is often low in unstressed plant cells. However, up to 580 µg/g FerT have been detected in roots of *Solanom melongena* (eggplant (Sun et al. 2015)) and up to 120 µg/g FerT in seeds of *Cannabis sativa* (hemp (Yamamoto et al. 1991)). The interest in tyramine-derived hydroxycinnamic acid amides arises significantly due to their diverse biological activities which comprise antioxidant, antidiabetic, anti-inflammatory, anti-cancer as well as cytotoxic, anti-melanogenesis and neuroprotective effects (Coman and Vodnar 2020). Their ability to quench reactive oxygen species (ROS) can be attributed to the chemical structure providing resonance stabilization via the extended p-orbitals (Gao et al. 2015). However, in many cases a clear mechanistic link between the reported positive effect in cell culture or animal models and a causative HCAA could not always be identified. At least in the case of antidiabetic activity, it was shown that a HCAA (coumaroyltyramine, CouT) inhibits α-glucosidases with an IC_50_ of 0.4 µM (Zhang et al. 2011). Therefore, co-consumption of carbohydrates with HCAA can lead to lower blood glucose levels (Holman et al. 1999). Another well documented target modulated by HCAA is tyrosinase. Murine B16 cells treated with FerT showed a significant reduction in melanin production due to down-regulation of gene expression of tyrosinase and related enzymes (Kim et al. 2018). In addition, active HCAAs containing extract from *Sophora japonica* exhibited tyrosinase inhibition in vitro (Lo et al. 2009). Despite being of high interest, the pharmaceutical and industrial applications of FerT are currently limited by the low yield of extraction processes from plants and the costly, fossil-based, multi-step processes involved in chemical synthesis (Roumani et al. 2020). However, biotechnological approaches to FerT synthesis offer a sustainable alternative. Two main production pathways are reported: either semi-synthetic methods that use enzyme catalyzed condensation in vitro, or whole-cell conversion processes. In one of the first reported semi-synthetic approaches, a lipase is used in a water-free reaction medium for the condensation of TyN and FerA (Alrub et al. 2012). In a 48-h reaction and with a six-fold excess of FerA over TyN, a yield of 93.5% was achieved. In an attempt with an ATP-dependent amide bond synthetase from *Azospirillum lipoferum* (*Al*CfaL)*,* best results were obtained with a large excess of TyN (50-fold) over FerA, yielding 76% FerT in a 48-h reaction (Zhao et al. 2025). A recent approach in a water-based system takes advantage of a promiscuous hydrolase/acyltransferase from *Pyrobaculum calidifontis* (PestE). In an acyltransferase reaction at pH 10, ferulic acid methyl-, ethyl- or vinyl esters serve as activated substrates in the transfer to TyN. In a 2-h reaction and a two-fold excess of ferulic acid methyl ester, up to 97% yield can be reached (Baumert et al. 2024). Clearly, enzymatic reactions in vitro are associated with drawbacks such as high cost for purified enzymes, cofactors, and organic solvents, which can be circumvented through biosynthesis cascades within living cells.

The first reported whole cell HCAA synthesis utilized a recombinant synthesis pathway of the key plant enzymes 4CL from *Arabidopsis thaliana* and THT from *Capsicum annuum* implemented in the production host *Escherichia coli (E. coli)* yielding ~ 189 mg/L (0.67 mM) of CouT, 135 mg/L (0.43 mM) FerT and 40 mg/L (0.13 mM) caffeoyltyramine (CafT) (Kang et al. 2009). In this report, the recombinant cells were supplied with HCAs and TyN. TyN can be readily produced from tyrosine via decarboxylation. Through further metabolic engineering, Sim et al*.* reported the construction of a strain that overproduces tyrosine and is equipped with tyrosine decarboxylase (TDC), along with 4CL and THT. This engineered system enabled TyN supply from endogenous tyrosine for the conversion of CouA to CouT, resulting in a final titer of 495.4 mg/L (1.75 mM; (Sim et al. 2015a)). The authors noted a strong correlation of tyrosine overproduction and CouT synthesis, indicating the importance of the chassis-strain performance in metabolic engineering.

**Fig. 1:**
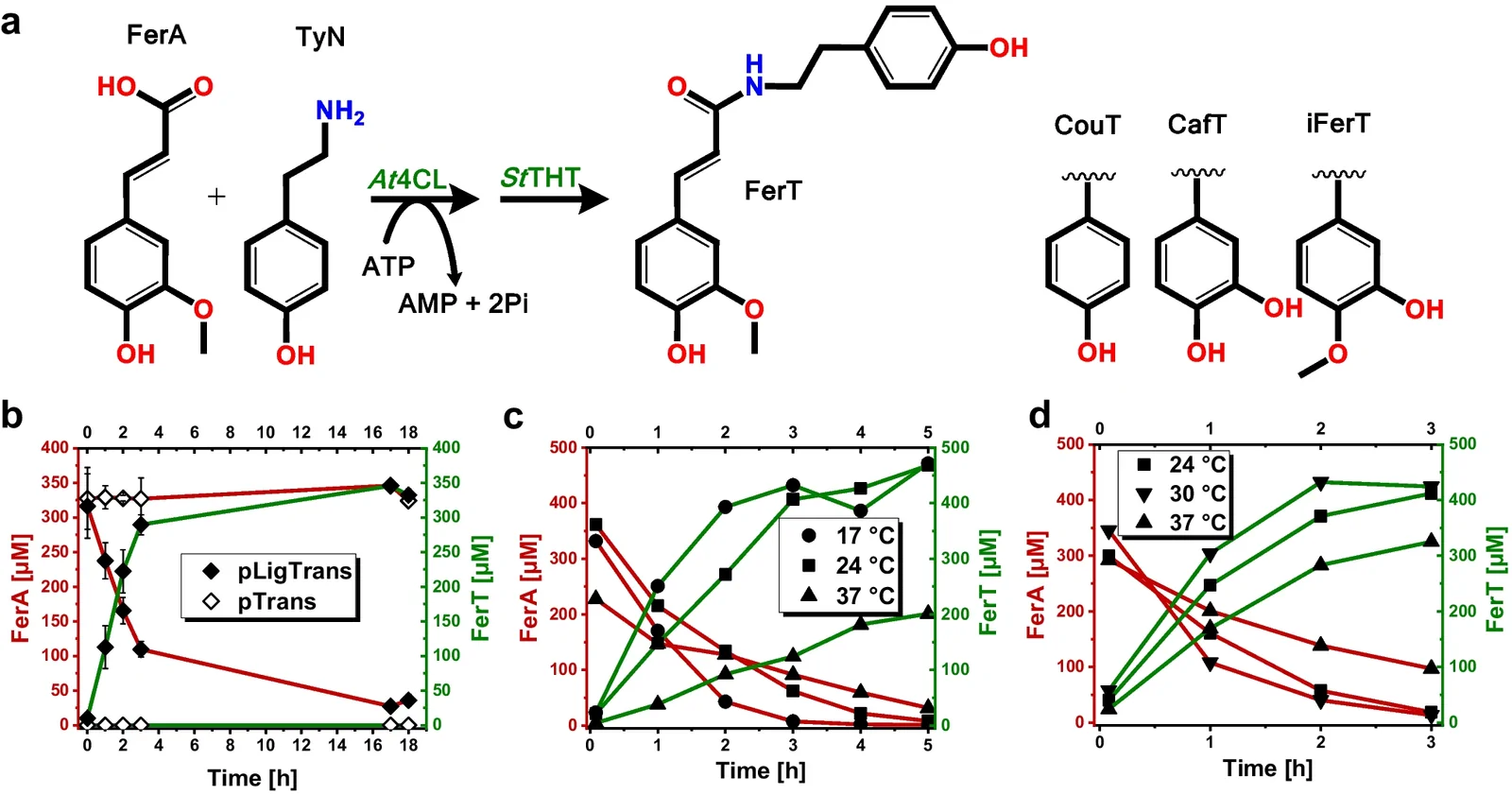
**a** Recombinant biosynthetic pathway to produce HCAAs. Ferulic acid (FerA) and tyramine (TyN) react in a two-step cascade by the plant derived enzymes *At*4CL and *St*THT (green) to feruloyltyramine (FerT). Coumaric-, caffeic- and isoferulic acid react to coumaroyl- caffeoyl- and isoferuloyl-tyramine (CouT, CafT and iFerT), respectively. **b** FerT production in *E. coli* BL21 ∆R, harboring either pLigTrans (*At*4CL and *St*THT, solid diamond or pTrans (*St*THT only, negative control, open diamond). *n* = 2 **c** FerT production in *E. coli* BL21 ∆R, pLigTrans, following recombinant protein expression at three different temperatures (17 °C, solid circle; 24 °C, solid square; 37 °C, solid up triangle) and production at 24 °C for 5 h. *n* = 1 **d** FerT production in *E. coli* BL21 ∆R, pLigTrans, following recombinant protein expression at 24 °C, and production at three different temperatures (24 °C, solid square; 30 °C, solid down triangle; 37 °C, solid up triangle) *n* = 1. Red lines: FerA consumption. Green lines: FerT production

The complex structure of many bioactive compounds, such as HCAAs, makes enzymatic synthesis an attractive alternative to classical chemical synthesis. In light of the urgent need to reduce dependency on fossil resources, water-based chemistry offers a green and carbon neutral alternative. Additionally, biocatalytically produced compounds are legally designated as natural, which adds value for certain markets, such as the food, feed and agrochemical industries. However, while more and more enzymatic activities are being described and many attempts are made to establish effective biosynthesis cascades, only a small fraction has reached a commercial stage (Choi and Lee 2023). The main reasons why biotechnological syntheses are often not competitive can be summarized as follows: (1) low product quantities, (2) elaborative product isolation, (3) long process times, and (4) complex process requirements. Individually or in combination, these factors significantly increase the cost of the end product, making it less competitive compared to conventional approaches (Patel et al. 2006). Hence, high product titers, short process times, simple process conditions, and easy downstream processing are key factors for establishing competitive biocatalysis (Porro et al. 2014). Although microbial production hosts have been shown to meet these criteria in some cases (Ewing et al. 2022), this is not universal. Many interesting and important secondary plant metabolites with phenolic character are not well tolerated by microbial production hosts, leading to low yields or the need for in situ extraction processes (Courdavault et al. 2021). While in situ extractions are technically challenging and expensive, more easy ways to counteract production limits are in high demand. Here, we show that TyN conjugates of hydroxycinnamic acids are easily accessible from soluble precursors FerA, CouA, CafA, isoferulic acid (iFerA) and TyN by a two-step synthetic reaction cascade (Fig. 1a) in bacterial whole cell catalysts. Most of the products exhibit a very low solubility in aqueous solution causing direct precipitation *in situ*. The equilibrium solubility of most of the target products is apparently below toxic concentrations for *E. coli* cells used for production. The precipitated products are easily separated from the fermentation broth by filtration and display a high degree of purity.

## Materials and methods

### Plasmid construction and *E. coli* strain

A pET28 expression construct coding for *St*THT, which was produced by subcloning from the vector pQE30 (Schmidt et al. 1999), as well as a pDEST-N112 expression construct coding for MBP-*At*4CL were kindly provided by the Leibniz Institute of Plant Biochemistry (Dippe et al. 2019). The coding region of MBP-*At*4CL was amplified by PCR and cloned via Golden Gate cloning with *Paq*CI (NEB) according to manufacturer’s protocol between the T7 promotor and the coding region of *St*THT, resulting in the bicistronic expression construct pLigTrans (plasmid map (Fig. S1) and sequence in supporting information (SI)). Transformation and cultivation of *E. coli* was performed according to standard protocols (Green and Sambrook 2012). “The *E. coli* BL21*ΔR* is derived from the wild type BL21 strain (Novagen) with an insertion of the functional part of DE3 in the *tyrR* gene (manuscript in preparation). The final genotype of the strain is: BL21 F^–^*omp*T *hsd*S_B_ (r_B_^–^, m_B_^–^) *gal dcm Δtyr*R::*lac*I*_*T7^RNAP^.”

### Shake flask experiments

BL21 *∆R*, pLigTrans were cultured in 300–500 mL baffled shake flasks containing 60–100 mL M9 media supplied with 1% (w/v) glucose (Roth) and 30 µg/mL kanamycin (Roth). Induction was performed at OD_600_ of ~ 0.6 with 0.5 mM IPTG (Roth). Following an indicated protein expression phase (~ 3 h) at a defined temperature, substrates were pulsed to 0.5 mM of FerA (Roth) and 0.5–0.6 mM TyN (abcr) to start the production phase. Cultures were incubated at the respective temperature with orbital shaking at 140–180 rpm (INFORS Multitron). OD_600_ was measured using UV/Vis-spectrophotometer (Thermo Fisher).

### Fermentations

Fermentation conditions have been adapted to standard high cell density fermentations (Knorre et al. 1991; Schaepe et al. 2014). All bioreactor experiments were performed in a DasGip® parallel bioreactor for microbial application equipped with online off-gas analyzer (Eppendorf AG) according to a standard procedure (see SI) with process parameters and experimental differences listed in Table 2. Briefly, the inoculum was prepared from a fresh M9-agar plate in the same day of inoculation, by maintaining cells in the exponential growth phase. The reactor vessel was filled with 350–500 mL M9-salts and supplied with 1×trace elements, 5 mM MgCl_2_, 0.2 mM CaCl_2_, three drops of antifoam (Struktol J647), 30 µg/mL kanamycin and 0.1% (w/v) glucose and inoculated to an OD_600_ of 0.02. Reactor temperature (37 °C), pH 7 (base control by 25% NH_3_-solution) and dissolved oxygen of more than 30% (DO, cascade of stirrer, airflow and O_2_) was maintained by online controllers (DasGip®). The process pH was kept at pH 7 for several reasons: (1) The neutrophilic *E. coli* grows optimally between pH 6 to 8 supporting high cell density fermentations and highest ATP yield per amount of glucose (Padan et al. 1981; Neidhardt et al. 1996). (2) The HCAs are less soluble at acidic pH (p*K*_a_ ~ 4.6) and TyN is less soluble at basic pH (p*K*_a_ ~ 9.7). At pH 7 the substrate HCAs and TyN are both soluble in high concentration (Table 3), enabling easy supply in (mixed) liquid form and simplifying technical regime from two to one pump, ensuring equimolar supply rate. (3) The HCAAs are poorly soluble at pH 7, supporting precipitation, omitting toxicity and facilitate product recovery. (4) Impurities in the precipitated product derived from unconverted substrates are most unlikely at pH 7. The bioreactors were operated using a pre-defined vessel script, dividing the fermentation process into phases and allowing extensive automation. A representative vessel script is given in SI. Glucose feed solution contained 30–50% (w/v) glucose, 5 mM MgSO_4_, 1×trace elements, 1×M9 salts. Substrate was supplied as mix of 120–400 mM FerA and TyN, pH 7.5 (NaOH). In case of iFerA pH was set to 9.2 (NaOH) and TyN (HCl) was fed separately. TyN was always used at 10% molar surplus over the HCAs, and the yield of conversion corresponds to the respective HCA. At OD_600_ of 15–20, cultures were supplied with a pulse of additional 5 mM MgSO_4_ and 0.2 mM CaCl_2_. The number of biological replicates is indicated in the figure description as *n*.

### HPLC analysis

Samples of cell suspensions were taken at indicated time points and diluted appropriately in 0.1 M CAPS pH 11, except for CafT (dilution in H_2_O), depending on the expected substrate and product concentrations. Samples were acidified by the addition of 2% formic acid (Merck) and extracted twice with equal volume of ethyl acetate (Fluka). Following phase separation by centrifugation (10,000 g, 5–10 min at room temperature), the organic phase was collected and evaporated using Savant™ SPD131DDA SpeedVac™ Concentrator (Thermo Fisher). Pellets were then resuspended in pure acetonitrile, sonicated (5 min at 22 °C, Sonorex Super 10 P BANDELIN) and then diluted with H_2_O to a final concentration of 10% ACN. In case of iFerT, the pellets were resuspended in 100% MeOH, sonicated as mentioned previously and diluted with H_2_O to 40% MeOH. Extracted samples were analyzed on a Shimadzu HPLC equipped with a 150× 4.6 mm YMC-Pack ODS-A, 12 nm pore, 5 µm bead (YMC Europe GmbH) at 30 °C, 1 mL/min flow rate, and detected at 310 nm. The mobile phase was mixed in a time dependent program between A: 12% ACN, 0.2% formic acid and B: 100% ACN. The gradient was as follows: 7 min 5% B, 3 min 10% B, 2 min gradient to 100% B, 2 min 100% B, re-equilibrated to 5% B. Calibrations were performed with authentic standards (listed in Table 1).

**Table 1:** Information about the standards used for calibration in this study. *NA* not applicable

Compound	Abbr	MW [g/mol]	ε [mol.L^−1^.cm^−1^]*	λ_max_ [nm]	Supplier
Ferulic acid	FerA	194.19	13,738	310	Roth
Coumaric acid	CouA	164,16	16,216	287	Roth
Caffeic acid	CafA	180,16	12,073	312	Roth
Iso-ferulic acid	iFerA	194,18	13,738**	310	BLD pharmatech
N-trans-feruloyl-tyramine	FerT	313,35	NA	NA	abcr/pure crystals
N-p-coumaryl-tyramine	CouT	283,32	NA	NA	MCE
N-trans-caffeoyl-tyramine	CafT	299,32	NA	NA	MCE
N-trans-isoferuloyl-tyramine	iFerT	313,35	NA	NA	AKos GmbH

^*^In phosphate buffer pH 7.5, (Kaeswurm et al. 2021)

^**^Due to structural similarity, the same *ε*_*λmax*_ as is used for FerA

### Purification of HCAAs by filtration and crystallization

HCAAs were collected by filtration using a 1 L filter head (DURAN®) and a VitraPOR borosilicate filter disc (ROBU®), por. 3 (16–40 µm) under vacuum pressure. Depending on its size, the filter cake was washed once or twice with 30–50 mL of 2% HCl, dried in desiccator, collected in falcon tube, and stored at −20 °C protected from light. Recrystallization was performed as described previously (Nomura et al. 2003; Tseng et al. 1992). Briefly, 1.5 g of filter cake of FerT was dissolved in 300 mL of ethyl acetate and filtered through a filter paper with vacuum filtration to remove undissolved powder. In a separatory funnel, the organic phase was washed with 30 mL of saturated NaCl solution. Following phase separation by gravity and discarding the water phase, the organic phase was dried with MgSO_4_ and filtered again. Ethyl acetate was evaporated using a rotary evaporator (Heidolph) at the recommended settings. The residue was dissolved in hot CHCl_3_: acetone 7:2 (v/v). After filtration, crystals were allowed to form at −20 °C for 3 days, filtered by vacuum filtration in cold room (4 °C) and washed with cold CHCl_3_. The pure crystals were dried in a desiccator and stored at −20 °C protected from light. Proton- and APT-NMR spectra were recorded to prove the identity of FerT (details in SI, Fig. S3).

## Results

### Production of FerT

For developing a biotechnological production process for FerT, we focused on a two-step reaction cascade catalyzed by the well-characterized 4CL2 from *Arabidopsis thaliana* (*At*4CL2, (Dippe et al. 2019)) and THT from *Solanum tuberosum* (*St*THT (Schmidt et al. 1999)). Both enzymes exhibit broad substrate specificity and substantial solubility upon recombinant expression, enabling the efficient synthesis of a diverse range of HCAAs. We designed an inducible expression vector coding for both *At*4CL2 and *St*THT in a bicistronic manner under the control of the T7 promotor. For improved recombinant expression, *At*4CL2 was cloned as *N*-terminal fusion with maltose-binding protein (MBP) and *St*THT as native gene, resulting in the plasmid pLigTrans (Fig. S1). pLigTrans was used to transform *E. coli* BL21*Δtyr*R (BL21 ΔR), a DE3 derivative. The transformed strain (BL21 ∆R, pLigTrans) was cultivated in 100 mL M9 medium at 37 °C. A strain expressing only *St*THT (BL21 ∆R, pTrans) served as a negative control and was treated similarly. Following isopropyl-*β*-D-thiogalactopyranoside (IPTG) induction, temperature was reduced to 24 °C for 3.5 h before the cultures were supplied with 0.5 mM FerA and 0.5 mM TyN. Culture supernatant was sampled and analyzed quantitatively by high-performance liquid chromatography (HPLC, for details see “Materials and methods”) for time-dependent change of FerA and FerT (Fig. 1b). We observed the formation of 300 µM FerT after 3 h and nearly complete conversion after 17 h. The time course of FerA depletion mirrors the product formation, while the FerA level remained constant in the control culture. The results confirmed that the expression construct is functional, the enzymes are active, the passage of substrates and product occurs inside and outside of the cell, and FerT exhibits reasonably high stability in the culture broth. Given that we focused on scaling the production, we went on to screen optimal expression and reaction conditions for the whole cell catalyst under investigation.

### Optimization of FerT production

In order to maximize FerT production, we analyzed the sensitivity of product formation with respect to the expression temperature of *At*4CL2 and *St*THT. The recombinant genes were expressed at 17 °C (16 h), 24 °C (5 h), or 37 °C (2 h), followed by a 5-h conversion phase at 24 °C, initiated by the addition of 0.5 mM FerA and TyN. For comparability we started the conversion phase with an equal amount of bio wet mass (BWM)*.* We found that the enzymes synthesized at 17 °C resulted in the highest initial product formation rate, followed by cells with recombinant expression at 24 °C with a slightly lower product formation. However, expression at 37 °C resulted in a considerably lower conversion rate and approximately two-fold lower product formation after 5 h (Fig. 1c). We concluded that the recombinant expression of at least one cascade gene is sensitive to temperature.

A similar biphasic setup was used to test sensitivity of product formation with regard to reaction temperature. Recombinant *At*4CL2 and *St*THT were expressed at 24 °C for 3 h, followed by a production phase at three different temperatures: 24 °C, 30 °C and 37 °C, during which FerA and TyN (0.5 mM each) were supplied. In contrast to the expected direct correlation between temperature and productivity, we observed that the highest temperature (37 °C) resulted in the lowest productivity. The reaction rate at 30 °C was the fastest, followed by the rate at 24 °C (Fig. 1d). Given the same start conditions, the decline in activity at 37 °C indicates a substantial lack of stability of the recombinant enzymes at that temperature.

### Expression temperature and protein stability

**Fig. 2:**
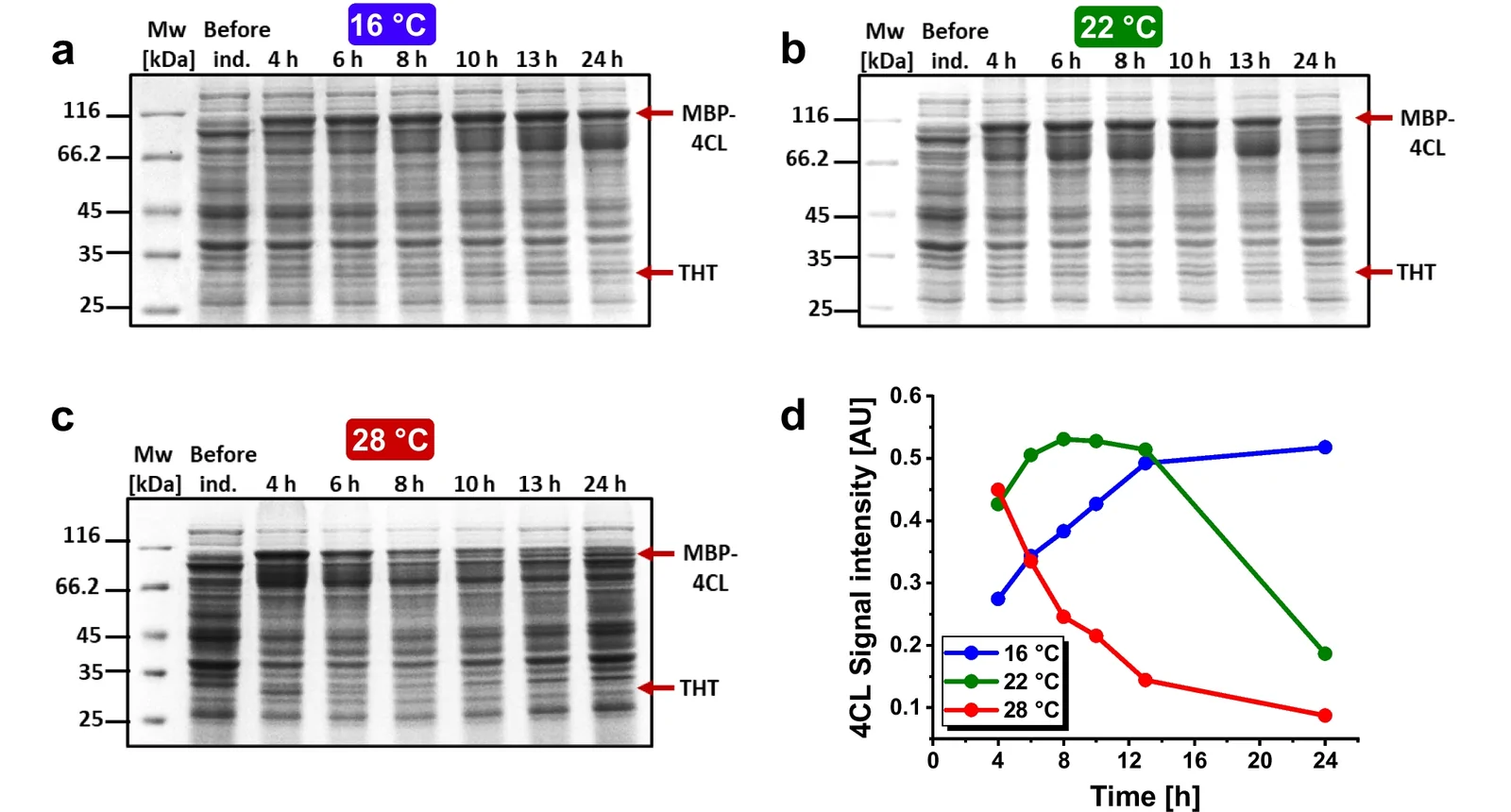
Expression pattern of *At*4CL2 and *St*THT at different temperatures. *E. coli* BL21 ∆R, pLigTrans was grown in M9 medium to OD_600_ 0.6 and induced with IPTG. The culture was split and incubated at either 16 °C (**a**), 22 °C (**b**) or 28 °C (**c**), for 24 h. MW_4CL_: 100 kDa. MW_THT_: 30 kDa (red arrow indicates electrophoretic mobility). **d** A densitometric analysis of PA-gels. Ind.: Induction with IPTG. *n* = 2

To characterize the kinetics of enzyme synthesis and potential degradation more precisely, we carried out test expressions at different temperatures and monitored time-dependent accumulation of recombinant proteins. Following IPTG induction of protein expression, a culture was divided into three subcultures, each incubated at either 16 °C, 22 °C, or 28 °C for 24 h. Samples were analyzed by Coomassie-stained sodium dodecyl sulfate–polyacrylamide gel electrophoresis (SDS-PAGE) (Fig. 2a–c). At 16 °C, the intensity of the band corresponding to *At*4CL2 was gradually increasing throughout the experimental time (Fig. 2d). In contrast, at 28 °C, there was a substantial initial accumulation of *At*4CL2 after 4 h of expression; however, the intensity of the band gradually decreased and almost vanished after 24 h. At 22 °C, the intensity of the band increased gradually up to 10 h, then decreased as well. Surprisingly, the intensity of the Coomassie-stained band corresponding to *St*THT coincides with band intensity of *At*4CL2, pointing to similar stability of *At*4CL2 and *St*THT. Attempts to maximize biotechnological production of FerT via those two enzymes therefore need to address the temperature-dependent turnover rates in process developments by adapted temperature profiles. Taken together, our tests indicate that application of *At*4CL2 and *St*THT for biotechnological production of FerT requires balancing different optimal temperatures to achieve high expression, high activity, and low degradation.

### Scaling FerT production

**Fig. 3:**
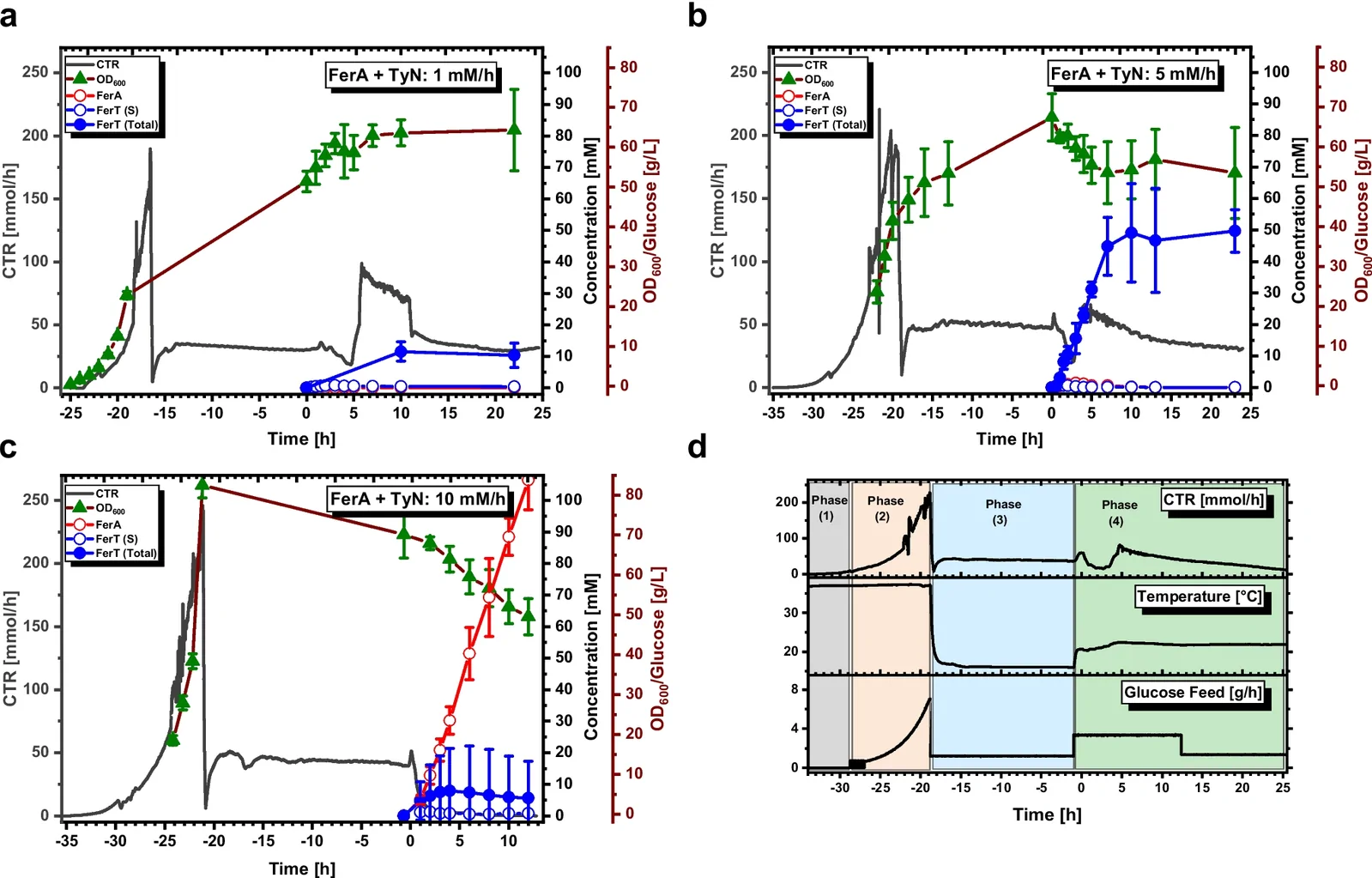
FerT production in *E. coli* BL21 ∆R, pLigTrans, following substrate supply rate of 1 mM/h (**a**), 5 mM/h (**b**) or 10 mM/h (**c**) for 10 h in 1 L bioreactor. For each set up *n* = 4 (Table 3). *t* = 0 h indicates the start of substrate supply. Dark gray solid line: online CTR data; red: [FerA] in mM; blue open circle: soluble [FerT] in mM; blue filled circle: total [FerT] in mM. **d** A representative set of processes data with color coded four phases: (1) batch phase at 37 °C (gray); (2) exponential growth phase at 37 °C (orange); (3) recombinant enzyme expression phase at 16 °C (blue) induced with IPTG or lactose and (4) production phase at 22 °C (green)

**Fig. 4:**
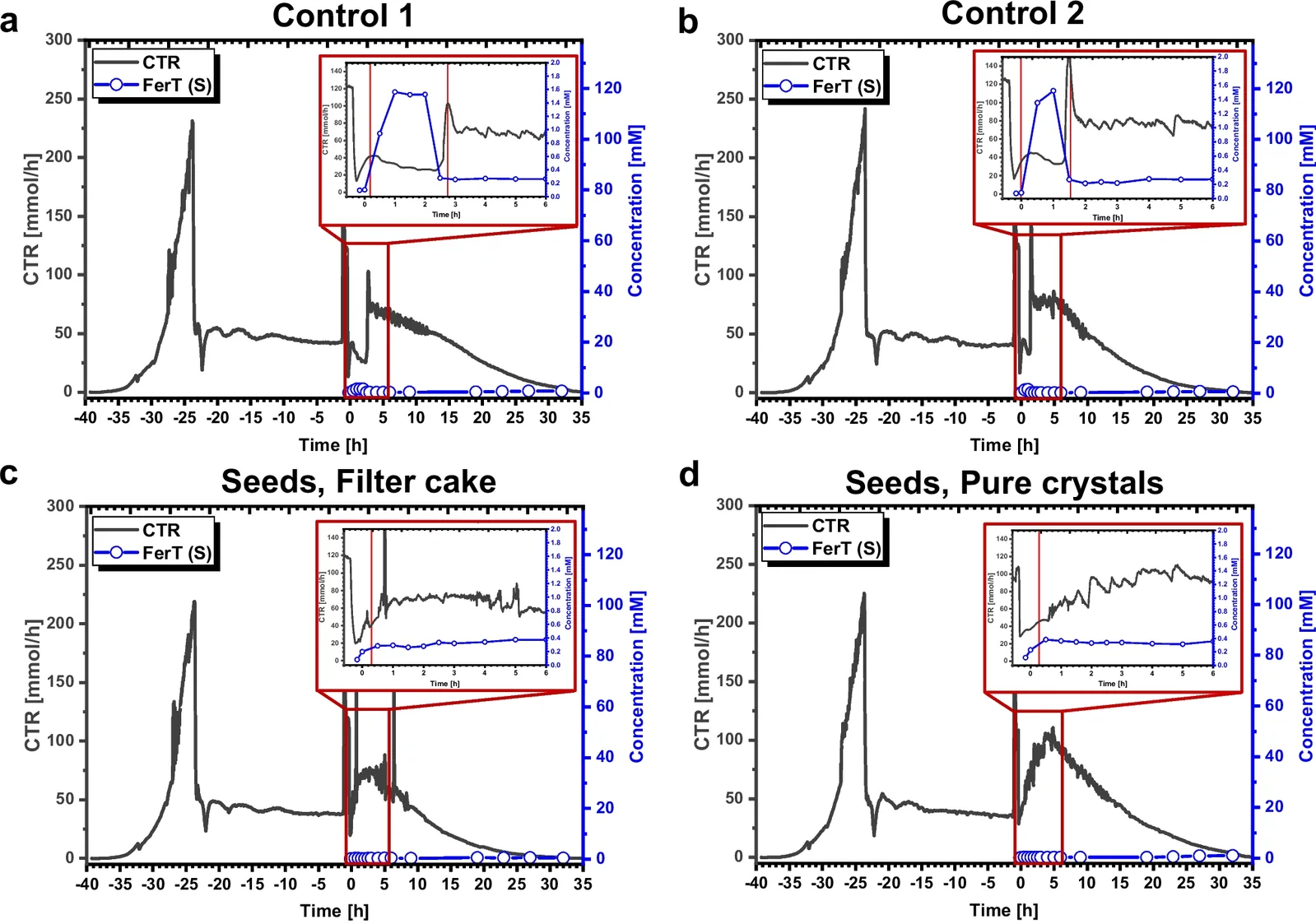
Testing the effect of seeds in FerT production. Recombinant protein expression was induced with IPTG in *E. coli* BL21 ∆R, pLigTrans and the production was carried out at 28 °C (*t* = 0 h). An initial drop in CTR (*t* = 0–3 h) is detected in the control experiments (**a**) and (**b**) (no seeds) which coincide with initial high soluble concentration of the product (FerT (S)). In seeded vessels (**c**) and (**d**), initial drop in CTR is also detected, however, soluble FerT level was below 0.4 mM and remained relatively constant. *n* = 1

For biotechnological production of FerT, economic considerations immediately dictate the transfer from shake flask experiments to a bioreactor setup. We followed the hypothesis that conversion rates are correlated to (1) enzyme concentration in the cell, (2) density of recombinant cells (equal to concentration of catalyst), and (3) conversion time. To maximize these criteria, we set up a multiphasic bioreactor process and tested 1, 5, and 10 mM/h substrate supply of FerA and TyN with a 10% molar excess of TyN (Fig. 3a–c). The process phases are sequentially divided into (1) batch phase, (2) exponential growth phase, (3) recombinant enzyme expression phase, and (4) production phase (see “Materials and methods”). The phase progression is either triggered by process values (dissolved oxygen (DO): phase 1 to 2, or feed value: phase 2 to 3) or set manually by change of temperature (phase 3 to 4). The glucose feed rate was kept limiting until the end of exponential growth (phase 2) to avoid overflow metabolism and acetate accumulation (Schaepe et al. 2014). In Fig. 3 the carbon transfer rate (CTR) shows the metabolic activity of the cells. The CTR rises from the beginning of the batch phase to the end of phase 2. After induction with IPTG and concomitant change in temperature from 37°C to 16 °C, the CTR immediately drops as expected due to the reduced metabolism. After the overnight expression phase, the temperature is set to 22 °C and the substrate supply is initiated at the indicated rate. At the beginning of phase 4, CTR briefly peaks in response to increasing temperature and, dependent on the supply rate of FerA + TyN, collapses thereafter completely. The optical density (OD_600_) increases from the inoculum of 0.02 to ~ 25 at the end of phase 2, to 50–60 at the end of phase 3 and did not substantially change in phase 4. Product formation was monitored by analyzing FerA and FerT by HPLC in phase 4 (see “Materials and methods”). Product concentrations in the supernatant of samples treated for 5 min at 10,000 g of all three supply rate set ups (Fig. 3a–c) remained much lower than the difference in supplied substrate vs. detected substrate. We assumed that the majority of the product precipitated due to low solubility of FerT (logP_OW_ 2.46, https://www.certara.com). Therefore, we diluted the total sample in 3-(cyclohexylamino)-1-propane-sulfonic acid (CAPS) buffer, pH 11, to facilitate dissolution of potential product precipitates by ionization. Analysis of diluted total sample showed indeed the instant and stochiometric conversion of FerA + TyN to 11.5 ± 3 mM FerT or 49.1 ± 15.6 mM in 10 h for substrate supply rates of 1 and 5 mM/h, respectively (Fig. 3a, b). Supply rates of 10 mM/h substrate mix resulted in rather a short production phase of 6 h ending with a maximum product concentration of 13.0 ± 5.9 mM FerT. Consequently, FerA concentration rises with 10 mM/h during the ongoing process (Fig. 3c). Each of the three different supply rates was done in four replicates with slightly different inducer and temperature settings (see Tables 2 and 2). The OD_600_ and product concentrations are given as averages of all 4 replicas in Fig. 3 (numbers are average ± SD of all four experiments). For clarity, the CTR data is a representative profile. The small deviations in OD and product concentrations highlight the strict process control by limited feed, allowing for very reproducible fermentation experiments. Overall, we noticed that a high supply rate of 10 mM/h led to a sustained decrease in CTR and concomitant loss in productivity. In contrast, in experiments with supply rates of 1 or 5 mM/h substrates, the CTR drops initially, but recovers within a time frame of 0.5–5 h (Fig. 3 and inlet Fig. 4a, b). Surprisingly, the temporary decline in CTR correlated strictly (*n* = 4/4) with the temporary high and apparently supersaturated concentration of FerT. With the beginning of substrate supply (phase 4), soluble FerT concentrations increased rapidly up to 2 mM and then dropped to around 0.2 mM concomitantly with the recovery of the CTR. We hypothesize that these high levels of soluble FerT are harmful to the cells, leading to reduced CTR until precipitation occurs and solubility equilibrium is reached.

**Table 2:** Process and experimental parameters of bioreactor conversions. *Conc.* Concentration, *Enz. Exp.* enzyme expression, *Ind.* Induction, *Sub.* substrate

Fermentation experiment	Conc. of Glu feed (w/v)	Start volume [mL]	Inoculum	Start OD_600_	Growth phase [h]	Mean OD_600_ at Ind	Enz exp. Phase [h]	Mean OD_600_ at production	Production phase [h]	Remarks
Fig. 3a: 1 mM/h	30%	500	8 mL of OD 29.35	0.47	7	22.8	18	51.4	23	Sub. feed stop 10h
Fig. 3b: 5 mM/h	50%	350	7 mL of OD 1.04	0.02	15	41	20	67	23	Sub. feed stop 10h
Fig. 3c: 10 mM/h			9.2 mL of OD 0.762	0.02	15.3	81.05	21	75	12	
Fig. 4: Seeding			4.5 mL of OD 1.48	0.02	15	46.98	25	72.88	35	
Fig. S2: 5 mM/h			5 mL of OD 1.35	0.019	16	50.93	19	70.1	26	
Fig. 6: 5 mM/h			5 ml of OD 1.4	0.02	13.4	55.5	20	75.35	10	CafT: feeding stopped after 2 h iFerT and TyN fed separately

**Table 3:** Source and concentration of substrates used in the fermentation experiments

Fermentation experiment	Brief explanation	HCA Source	TyN Source	Concentration of substrate stock [mM]
Fig. 3a: 1 mM/h	IPTG/Lactose, 22/28 °C	Roth	abcr	FerA: 120
Fig. 3b: 5 mM/h	IPTG/Lactose, 22/28 °C	Sigma Aldrich	abcr	FerA: 300
Fig. 3c: 10 mM/h	IPTG/Lactose, 22/28 °C	BLDpharm	abcr	FerA: 400
Fig. 4: Seeding	IPTG + 28 °C	BLDpharm	ZellX Biochem	
Fig. S2: 5 mM/h	IPTG/Lactose, 22/28 °C	BLDpharm	BIOSYNTH s.r.o	
Fig. 6: 5 mM/h	IPTG + 28 °C, iFerA, CouA, CafA	Table 1	BIOSYNTH s.r.o	CafA: 430; iFerA: 371; CouA: 468

Throughout the experiments, we observed a strong correlation between productivity and CTR values, suggesting that productivity of the process might be further enhanced through improvement of CTR values in phase 4. One optimization could be achieved by avoiding the temporary decline in the CTR, putatively in response to the supersaturation of FerT. We speculated that seeding FerT crystals at the beginning of phase 4 might circumvent the temporary decline in the CTR. In a set up with four parallel bioreactors with a similar parameter setting as in Fig. 3b with a substrate supply rate of 5 mM/h, two reactors served as controls (without seeds). The third reactor was seeded with crude precipitate (filtrate) from a previous experiment (783 mg corresponding to 5 mM FerT) and the fourth reactor was seeded with pure crystals of FerT obtained from a multi-step purification (783 mg, see below). In the control experiments, we observed again the temporary decline in CTR for 2.5 h (Fig. 4a) or 1.5 h (Fig. 4b) before CTR instantly recovered (Fig. 4a, b inlet). In the processes containing FerT seeds (crude or pure), we also observed a drop in CTR upon start of substrate supply but a constant recovery of CTR within 1 and 3 h (Fig. 4c, d), concluding the seeds did not prevent the initial drop in CTR but supported faster recovery. Interestingly, in the processes spiked with FerT seeds, the FerT concentration in the supernatant remained below 0.4 mM, unlike the control reactions, which temporarily exhibited a concentration of 1.4 mM FerT in parallel with the transient drop in CTR. The overall product formation was limited by a slow decline of CTR over 32 h of phase 4 with product formation falling short to supply rate at around 19 h, leading to a maximum of 108 ± 7.6 mM (33.9 g/L) FerT in all four bioreactors. In conclusion, this experiment unveils that seeding FerT triggers a faster recovery of CTR and avoids supersaturation of FerT. However, FerT seeding did not prevent the initial drop in CTR upon supply of substrates, did not change the final product amount, and resulted in a less filterable precipitate that easily clogged the filter compared to the non-seeded broth.

### Operational robustness in response to inducer and temperature

**Fig. 5:**
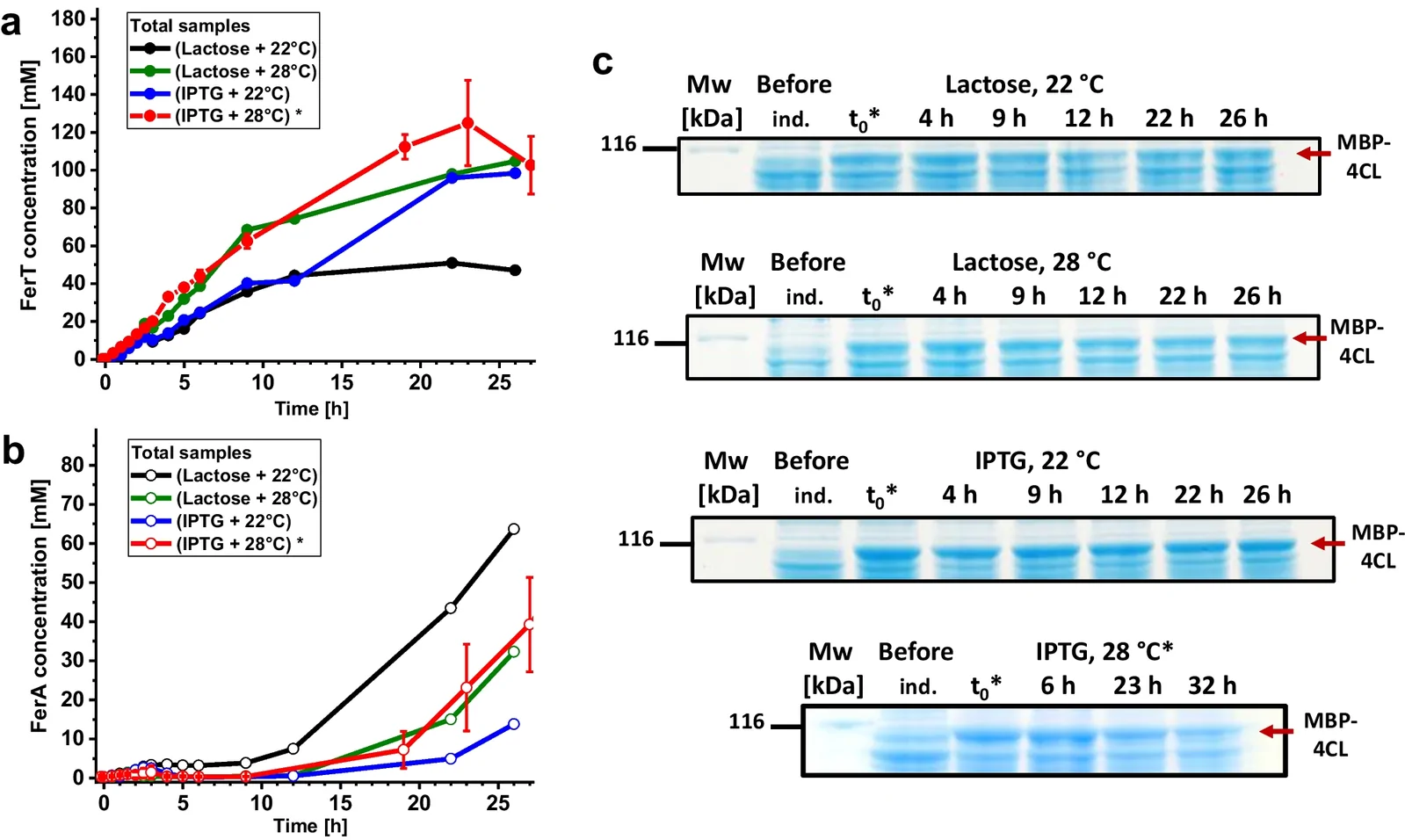
FerT production at four different conditions. Substrate supply rate is 5 mM/h. **a** and **b** FerT and FerA concentrations in total samples. **c** SDS-PAGE analysis of *At*4CL2 expression. t_0_ indicates the start of production. *n* = 1. Results of the settings IPTG, 28 °C were obtained from seeding experiment (Fig. 4a, b, *n* = 2)

In pursuit of a sustained metabolic activity in the production phase, we additionally examined induction by lactose, a cost-effective alternative to IPTG for large-scale processes (Kilikian et al. 2000). Considering two temperatures at the production phase to find a balance between enzyme productivity and stability, three conditions were set up in a parallel experiment: (1) IPTG + 22 °C, (2) lactose + 22 °C, and (3) lactose + 28 °C. The results of the 4th condition (IPTG + 28 °C) were obtained from the previously mentioned experiment (seeding). Substrate supply rate was set to 5 mM/h and continued until CTR was below 10 mmol/h in all vessels. As expected, lactose induction resulted in a lower protein expression compared to IPTG. Additionally, a slight decline in protein band intensity was observed at 28 °C compared to 22 °C (Fig. 5c). Interestingly, the initial rate of production (0–3 h) was relatively similar in all conditions, despite the low expression after lactose induction and the different reaction temperatures (Fig. 5a), clearly indicating that recombinant enzyme activity is not the rate limiting factor for product formation in this setting. Production rate was slightly faster at 28 °C in between 3 and 10 h; however, the final product concentration was relatively similar for three conditions (102 ± 3 mM, 31.9 g/L, 26 h). The combination of lactose induction and production at 22 °C resulted in the lowest product formation (47 mM, with stagnation of the product formation at ~ 12 h). Whether this is statistically significant needs to be determined with additional fermentations. Up to 9 h of the production phase, the FerA level remained around 0 mM (Fig. 5b), indicating a rapid and immediate consumption of substrates, with substrate supply being the rate limiting factor. However, FerA concentration starts to increase in the ongoing process, and, alongside this, the production rate of FerT gradually declines. These results indicate that total product formation is limited by a critical drop of CTR and is not limited by the recombinant enzymes (Fig. S2). To conclude, we observed that a higher conversion temperature resulted in a higher rate of production, and therefore, recommended for shorter duration. Similar results can be obtained at lower production temperatures for extended duration. Lactose induction results in similar production as IPTG induction if the reaction was carried out at 28°C, and therefore, it could be considered as a suitable alternative under these conditions. Variations in temperature and inducer type had minor impact on the final product titer, highlighting the robustness of the process under parameter fluctuations. In four out of five fermentations (excluding the lactose+22 °C setup), the mean final titer was 99.47 ± 7.64 mM. When all five fermentations were considered together, the mean titer decreased slightly to 89.36 ± 22.36 mM. However, in all cases, the loss of viability indicated by a substantially reduced CTR value coincides with a stop of product formation and should therefore be considered as the causal reason for the limit of production.

### Formation of coumaroyltyramine (CouT), caffeoyltyramine (CafT) and isoferuloyltyramine (iFerT)

**Fig. 6:**
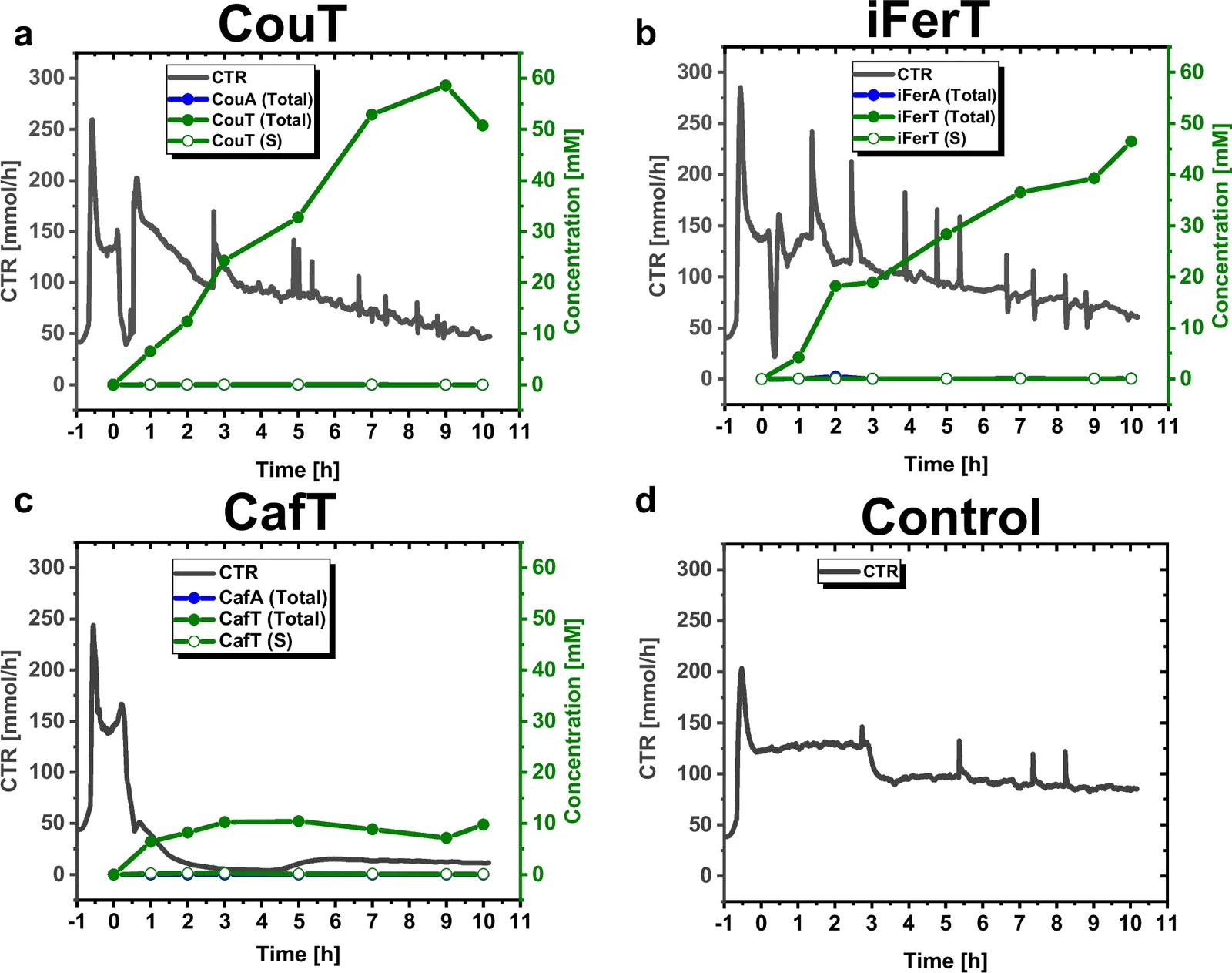
Production of CouT (**a**), iFerT (**b**), and CafT (**c**) in *E. coli* BL21 ∆R, pLigTrans. Fermentation set up as in Fig. 3b. t = 0 h indicates the start of substrate supply (5 mM/h). Dark gray solid line: online CTR data; blue filled circle: substrates [CouA, iFerA or CafA] in mM; green filled circle: total products [CouT, iFerT and CafT] in mM; green open circle: soluble products [CouT, iFerT and CafT] in mM. **d** Control vessel showing CTR of the producer strain by omitting the substrate supply. *n* = 1

*At*4CL2 and *St*THT have been shown to be able to accept a variety of either carboxylic acid substrates (Dippe et al. 2019) or CoA esters (Schmidt et al. 1999). Hence, we evaluated the possibility to produce a set of alternative products under the established process conditions by substituting FerA by iFerA, CouA or CafA. Four parallel bioreactor processes were set up with 5 mM/h substrate supply rate of CouA + TyN, iFerA + TyN, CafA + TyN, and a control without substrate supply. Due to solubility limitations of iFerA and volume limitations in the reactor, the maximum total substrate added was 50 mM. After 10 h of production at 28 °C, we reached final concentrations of 50.7 mM CouT (14.4 g/L, yield ~ 100%), 46.5 mM iFerT (14.6 g/L, yield ~ 93%) and 9.7 mM CafT (2.9 g/L, yield ~ 20%, Fig. 6a–c). While the production of CouT and iFerT was well tolerated and only ceased due to substrate limitation, the CTR in the CafA setup collapsed to 4 mmol/h and only recovered after 5 h, remaining very low afterwards. We concluded that the resulting product CafT is toxic to the production host. In the control set up (Fig. 6d), the CTR remains stable throughout the production phase, proving that the production strain is not metabolically compromised by the recombinant expression of *At*4CL2 and *St*THT. The step of CTR from 120 to 100 mmol/h at around 3 h corresponds to the transition from batch glucose consumption to limited fed batch conditions. Glucose feed rate in the expression phase was set slightly above the maximum consumption rate to avoid limitations in recombinant expression.

### FerT purification and NMR analysis

**Fig. 7:**
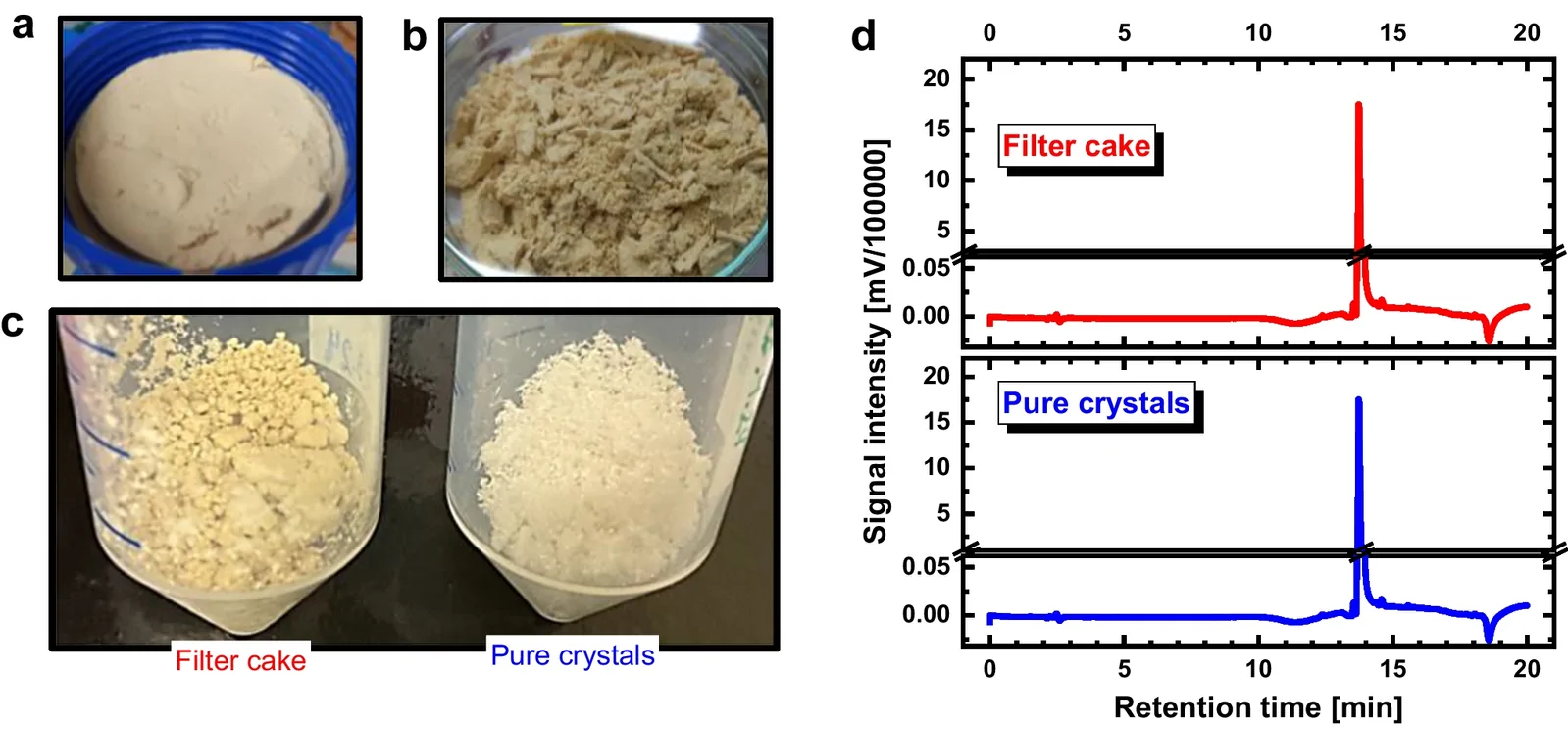
FerT purification. FerT was retrieved by filtration (16–40 µm glass filter), washed with 2% HCl and purified by crystallization. **a** Filtered cake over filter disk. **b** Product collected from 1 L reactor (IPTG + 28 °C, 5 mM/h substrate supply rate, ~ 22.75 g. Figs. 4a and 5a, red line). **c** and **d** Comparison between product appearance and purity (HPLC analysis) before and after crystallization, respectively. Samples for HPLC analysis were prepared by dissolving the product in 50% MeOH and diluting in ddH_2_O to the same final concentration

As described earlier, FerT precipitated during the fermentation process due to its low solubility in water. This physical property greatly facilitated the purification process from the fermentation broth. We developed a protocol in which crude fermentation broth is filtered through a glass filter with porosity 3 (16–40 µm) and washed the filter cake with 2% HCl (Fig. 7a–c). The majority of FerT was retained on the glass filter and only 0.44% was found in the flowthrough. The filter cake was further purified by recrystallisation from chloroform:acetone (7:2) mixture. HPLC analysis of the filter cake sample and recrystallized FerT is shown in Fig. 7d. Strikingly, the two chromatograms show a single FerT peak and the lack of detectable contaminants above 0.01% total peak area. Mass balance of filter cake material indicated an overall purity of greater than 95%. The recrystallized FerT was analyzed by NMR to confirm its identity and purity (NMR data in Fig. S3). No impurities other than the solvents used in the recrystallization were present in the NMR spectra. Taken together, the precipitated FerT can be readily obtained by filtering the fermentation broth with exceptional high purity of more than 99%.

## Discussion

Our investigation focuses on developing a scalable biotechnological process for the economical production of HCAAs in *E. coli.* Compared to previous studies (Kang et al. 2012; Sim et al. 2015b), we concentrated on improving expression conditions of the plant-derived catalysts at close to high-cell-density fermentation (Shiloach and Fass 2005). Our results demonstrate the need to balance process temperature between soluble expression, longevity and activity of recombinant catalysts. In contrast to a simultaneous expression and reaction phase, which requires a compromise between expression temperature and reaction temperature, the biphasic process of sequential expression and subsequent reaction phase offers superior productivity, particularly for critical enzymes frequently encountered in the plant kingdom (Back et al. 2001; Dippe et al. 2019; Wang et al. 2011). In addition, decoupling the expression and reaction phases may also be advantageous if the biosynthesis reaction impairs growth to such an extent that high cell densities and thus high catalytic rates of product formation can no longer be achieved (Wallace 2004). Furthermore, the best theoretical situation for product formation is to channel the total carbon flux in the direction of product formation (Lee and Wendisch 2017), eighter directly by transferring substrate carbon to product carbon, or indirectly by supplying metabolic energy for cofactor regeneration, membrane potential driven transport events and many more. Hence, decoupling growth and production can offer superior yields on carbon substrate. In our study, we used glucose primarily to maintain the basic metabolism while supplying the substrates HCAs and TyN for enzymatic conjugation. In principle, HCAAs can be produced via metabolic engineering by leveraging a tyrosine-overproducing host strain to enable both tyrosine decarboxylation to TyN (Poethe et al. 2024; Ma et al. 2025) and tyrosine conversion to FerA (Li et al., 2025). However, efficient implementation of these enzyme cascades remains to be demonstrated. Currently, HCAAs are only available in limited quantities from plant extraction or via costly chemical synthesis, rendering them prohibitively expensive (e.g., FerT: €50,000/g, Sigma-Aldrich). In contrast, TyN is accessible through the decarboxylation of tyrosine, a reaction readily implemented in engineered tyrosine producers. TyN is commercially available at ~ €4/g (vs. ~ €0.5/g for tyrosine, Sigma-Aldrich), making it only marginally more expensive. Biotechnological FerA production is reported from engineered microbial strains with titers of up to 5 g/L (Zhou et al. 2022). That would not be sufficient to fully exploit the HCAA synthesis process described here and would result in a lower product concentration. However, FerA is commercially available at ~ €1.5/g (Sigma-Aldrich). Taken together, the condensation reaction generates currently a massive increase in value. Despite that, substantial cost reductions are achievable by streamlining the substrate supply chain, starting from tyrosine as a central precursor for both TyN and FerA. The most elegant goal is to engineer a microbial host capable of overproducing tyrosine from inexpensive carbon sources, thereby enabling efficient, cost-effective, and scalable biosynthesis of HCAAs.

One key aspect in evaluating the fermentation parameter in regard to performances is the CTR in the production phase. As an online measure of direct ongoing carbon oxidation (Royce 1992), it allows a close inspection of the effect of product formation on the host metabolism. Cells that have undergone the expression phase at low temperature and were shifted back to the reaction temperature of 28 °C show a continuous CTR value of ~ 100 mmol/h, which correlates well with the continuous glucose feed of 16 mmol/h when not supplemented with reaction substrates (Fig. 6d). In contrast, cells that undergo product formation show a continuous decline in CTR highlighting that product formation is exhausting. When the CTR falls below a critical value, substrate starts to accumulate showing that the product formation rate is slower than the supply rate. Rather than only analyzing product concentration and cell density (Patnaik et al. 2008), the CTR gives direct insights into causal reasons for the loss of productivity by visualizing changes in metabolism (Heyman et al. 2020). A long lasting low CTR in addition to glucose accumulation most likely indicates a collapsed metabolism concomitant with a shortage of cofactor regeneration. Besides the low CTR at the end of the production phase, we observed a tight correlation of supersaturated concentrations of FerT and a 50% to 60% drop in the CTR once the substrate supply starts (Fig. 4a, b and Fig. 6). We assume that HCAAs are toxic to *E. coli*, making their concentration a critical parameter for viability and thus resulting in an inverse correlation between CTR and HCAA concentration. Some HCAAs have been tested for antimicrobial activity against different Gram-positive strains. The minimal inhibitory concentration (MIC) was found to be in the range of 0.3–1.5 mM for FerT and CafT (Georgiev et al. 2013). For Gram-negative bacteria MICs have not been reported so far. The low apparent equilibrium concentration of ~ 0.2 mM FerT in the later phase of production (> 4 h, Figs. 4, 5, S3) is presumably advantageous to ensure an ongoing metabolism, allowing for a robust ATP-dependent FerA-ligation and transfer reaction. Also, CouT and iFerT show a relatively low apparent equilibrium concentration of ~ 0.2 mM CouT and 0.1 mM iFerT, allowing the CTR to recover after the initial drop (Fig. 6a, b). However, for CafT the apparent equilibrium concentration is slightly higher with ~ 0.6 mM leading to the effect that the CTR does not recover during the production phase (Fig. 6c). Process modifications that reduce CafT solubility, such as lowering the pH to a more acidic range or adding catechol-complexing agents, may enable recovery of the CTR and enhance CafT production. These strategies have not yet been experimentally tested. Alternatively, CafT might exert higher toxicity to the production host due to its catechol moiety compared to CouT, FerT and iFerT (Schweigert et al. 2001). However, the rapid response of CTR to the addition of substrates (10 min from 120 to 20 mmol/h) may suggest that the cells require an adaptation to the supplied compounds. This is supported by four findings: (1) At the beginning of the production process the soluble concentration of FerA, CafA, and CouA is far below the reported minimal inhibitory concentration (MIC) of 9 mM FerA, (Borges et al. 2013), 39 mM CafA, (Andrade et al. 2015), and 0.75 mM CouA, (Sánchez‐Maldonado et al. 2011), making it unlikely to exert a substantial toxic effect. (2) At a supply rate of 1 mM/h FerA, no significant decrease in CTR was observed even at product amounts of 10 mM FerT. (3) At a higher supply rate of 10 mM/h FerA no recovery of the CTR was detected, leading to the assumption that higher concentrations presumably overload the adaptation process. (4) Seeding crystals of FerT at the beginning of production phase prevents over-saturation of FerT but not the initial drop in CTR (Fig. 4c, d), speaking against a substantial harmful effect of the product FerT. Further experiments can clarify if modulated substrate supply rates can stabilize the CTR to maintain high metabolic activity.

One of the main bottlenecks in the commercialization of biotechnologically produced compounds is the laborious, hence expensive, recovery from the fermentation broth (Harrison et al. 2015). Here, we show a surprisingly simple recovery strategy that already yields very pure product. The purity is most likely driven by the use of a defined salt medium and the better solubility of potential impurities like the reaction substrates FerA and TyN. The recovery of phenolic compounds such as vanillin, FerA, gallic acid, and polyphenols including flavones, catechins, and anthocyanins from fermentation broths has been explored by a range of separation strategies (Díaz-Montes and Castro-Muñoz 2022; Huang et al. 2008). Two-phase liquid–liquid extraction remains a widely studied method (Zhang et al. 2025), with vanillin effectively extracted in solvents such as ethyl acetate or ionic liquids, offering good selectivity and scalability (Paul et al. 2021; Rawat et al. 2022). However, solvent toxicity, emulsion formation, and additional solvent-removal steps limit its industrial appeal. Adsorption on polymeric resins or functionalized beads (e.g., XAD resins for gallic acid or flavonoids) provides high binding capacity and reusability, while enabling continuous operation, although desorption efficiency, fouling, and resin regeneration costs remain challenges (Kammerer et al. 2014). Precipitation techniques, such as calcium salt precipitation of gallic acid or pH-shift-induced crystallization of FerA, are inexpensive and easy to implement, however, often lack specificity, e.g., by co-precipitation of sugars, proteins, or other metabolites (Shi 2016). Overall, a final filtration step of precipitated product is not selective for the target product and runs the risk of clogging the filter. We observed different efficiencies of the filter process for to the HCAA products. While FerT and iFerT could be filtered well, CouT and CafT lead to substantial filter clogging when using filters with a pore size of 16–40 µm. We speculate that the particle size and shape of the different products differs and we will address this question in future experiments, e.g., via microscopy. In addition, fermentation broth stored for more than two days at 4 °C also substantially impaired filtration. However, in comparison to the downstream processing methods currently used for the isolation of polyphenolic compounds from fermentation broth, the filtration method disclosed here offers outstanding economic and ecological advantages.

## Supplementary Information

Below is the link to the electronic supplementary material.

ESM 1 Supplementary Material 1 (DOCX 222 KB)

## Data Availability

No datasets were generated or analysed during the current study.
