# Correction: Efficient biotechnological production of *N*-(Hydroxycinnamoyl) tyramines by utilizing plant-derived enzymes in a bench-scale bioreactor with in situ product precipitation

**DOI:** 10.1007/s00253-026-14044-0

**Published:** 2026-09-19

**Authors:** Israa Abuobida Mohamed, Qais Al-Khawlani, Raika Milde, Niels Valentin Heise, Martin Dippe, Mohamed Nagia, Ludger A. Wessjohann, Markus Pietzsch, Bodo Moritz

**Affiliations:** 1 Institute of Pharmacy, Martin-Luther-University Halle- Wittenberg, Weinbergweg 22, 06120 Halle (Saale), Germany; 2 Leibniz-Institute of Plant Biochemistry, Department of Bioorganic Chemistry, Weinberg 3, 06120 Halle, Germany; 3 Institute of Chemistry, Martin-Luther-University Halle-Wittenberg, Kurt Mothes Straße 2, 06120 Halle, Germany; 4 Department of Chemistry of Natural Compounds, Pharmaceutical and Drug Industries Research Institute, National Research Center, El Buhouth St. 33, 12622 Cairo, Egypt

**Correction to: Applied Microbiology and Biotechnology**

**https://doi.org/10.1007/s00253-026-13997-6**

In this article, the author’s name Mohamed Nagia was incorrectly written as Mohammed Nagia.

The original article has been corrected.

