# Supplementary material for: Efficient biotechnological production of N-(Hydroxycinnamoyl)tyramines by utilizing plant-derived enzymes in a bench-scale bioreactor with in situ product precipitation
Source: Appl Microbiol Biotechnol. 2026 Aug 26;110:258. doi: 10.1007/s00253-026-13997-6 (PMC13518753; doi:10.1007/s00253-026-13997-6)
Supplement: Supplementary file 1 [file 253_2026_13997_MOESM1_ESM.docx]

**Supporting information:**

**Fig. S1:** Plasmid map of pLigTrans. Coding genes of lacI MBP_4Cl and THT are indicated. Bicistronic mRNA synthesis is driven by T7 promotor. Translation is facilitated by strong RBS 5’ to MBP_4Cl and THT. The plasmid carries kanamycin resistance (KanR) and pBR322 origin of replication.

Figure S2

**Fig. S2:** CTR and FerT correlation to inducer and temperature variation. FerT production in *E. coli* BL21 ∆R, pLigTrans, following substrate supply rate of 5 mM/h. Process set up as in Fig 3b. with indicated differences of inducer IPTG (c, d) and lactose (a, b) and reaction temperature of 22 °C (a, c) and 28 °C (b, d). t = 0 h indicates the start of substrate supply. Dark gray solid line: online CTR data; red: [FerA] in mM; blue: total [FerT] in mM, pink triangle: glucose in g/L; green triangle: OD_600_. *n* = 1.

Figure S3: Atom assignment of FerT for NMR.

Results of ^1^H and ^13^C NMR:

1H NMR (502 MHz, DMSO-d6) δ 9.39 (s, 1H, OH’), 9.15 (s, 1H, OH), 7.96 (t, J = 5.6 Hz, 1H, NH), 7.31 (d, J = 15.7 Hz, 1H, 11-H), 7.11 (d, J = 1.7 Hz, 1H, 13-H), 7.01 (d, J = 8.4 Hz, 2H, 3-H, 5-H), 6.98 (dd, J = 8.2, 1.7 Hz, 1H, 17-H), 6.79 (d, J = 8.1 Hz, 1H, 16-H), 6.68 (d, J = 8.3 Hz, 2H, 2-H, 6-H), 6.43 (d, J = 15.7 Hz, 1H, 10-H), 3.80 (s, 3H, 18-H), 3.33 (dd, J = 13.3, 6.5 Hz, 2H, 8-H), 2.65 (t, J = 7.4 Hz, 2H, 7-H).

13C NMR (126 MHz, DMSO-d6) δ 165.29 (C-9), 155.61 (C-1), 148.21 (C-15), 147.80 (C-14), 138.83 (C-11), 129.51 (C-4), 129.44 (C-3, 5), 126.44 (C-12), 121.48 (C-17), 119.04 (C-10), 115.64 (C-16), 115.10 (C-2, 6), 110.76 (C-13), 55.52 (C-18), 40.64 (C-8), 34.42 (C-7).

Proton- and APT-NMR spectra were recorded using the Varian spectrometers (Darmstadt, Germany) VNMRS (500 MHz). The sample (40 mg) was dissolved in DMSO-d6. Chemical shifts are reported in parts per million referenced to the solvent signal. For NMR signal assignment standard 2-D NMR experiments, (gDQCOSY, gHSQCAD_PS, gHMBCAD) were carried out using the VNMRJ 3.2 pulse sequences. Data processing was performed with MestreNova software (Mestrelab Research, La Coruña, Spain), respectively. 3J coupling of 10 and 11 with 15.7 Hz typical for trans-double bonds.

**Supporting Methods**

**Parallel fermentation:** All bioreactor experiments were performed in a DasGip® parallel bioreactor for microbial application equipped with online off-gas analyzer (Eppendorf AG). Pump flow rates for glucose feed and substrates are calibrated with the respective liquids at 10 ml/h before each run. Pumps for base (25 % NH_3_) and antifoam (20% Struktol® J647) are calibrated with water. The assembled bioreactors were filled with 300-500 mL of 1x M9 salt medium (Table S1) and autoclaved. Before connecting the bioreactors to the system, the M9 medium was adjusted to 5 mM MgSO_4_, 0.2 mM CaCl_2_, 1 X trace elements (Table S2), 30 µg/ml kanamycin and 3 drops of 50 % Struktol® J647. After connecting the vessels, the pO_2_ electrodes were calibrated to 100 % dissolved oxygen by saturating each vessel with air at 37 °C, 40 sL/h gassing, 200 rpm stirring. Absence of oxygen (0 % dissolved oxygen) was adjusted with pure N_2_ under the same conditions. The glucose feed solution was prepared by adjusting autoclaved 30 % (final w/v) glucose to 1 x M9 salts (Table S1), 10 mM MgSO_4_, 1 x trace elements (Table S2). Substrates were prepared by dissolving a HCA and TyN as molar mix of 1:1.1 by adjusting pH to 7.5 (HCl). iFerA was dissolved individually by adjusting pH to 11. The pH was automatically controlled by adding NH_3_-solution (25 %) to the process when required. Process parameters were programmed to maintain pH 7, 37°C and dissolved O_2_ level ≥ 30 % air saturation. Initial batch glucose concentration was 0.1 % (w/v) and the process was started with an initial inoculum of log phase growing cells to OD_600_ = 0.02. The vessel script provided automated control of process parameters and phase progression (a representative vessel script is given below). The process was divided in three main phases: I) initial batch phase; cells grow under substrate excess. Depletion of substrate causes a dissolved oxygen peak and trigged phase progression. Phase II) Exponential fed-batch phase. Cells grow under substrate limitation with *μ* of 0.4 with an initial glucose feed of 0.8 ml*h^-1^. At 14.6 mL*h^-1^ glucose feed phase III (production phase) is initiated with constant feed. With the beginning of phase III lactose (0.5 % mM) or IPTG (0.5 mM) is added and temperature is set directly (IPTG induction) or 2 h after addition of lactose to 16 °C (lactose is consumed in between the first and second hour after addition). Next morning (~20 h after induction) vessels are equilibrated to 22°C or 28°C for 1h and substrate supplementation is started (production phase t= 0 h) with indicated rate. Samples for analyses such as SDS-PAGE and HPLC were taken at regular intervals and flash frozen in liquid nitrogen. Glucose concentrations were determined by glucose analyzer (YSI).

**Table S1:** M9 salt solution fivefold concentrated.

| **5 x M9-Salts** | Component | Concentration [M] |
| --- | --- | --- |
|  | (NH_4_)_2_SO_4_ | 0.093 |
|  | NH_4_Cl | 0.0465 |
|  | NaH_2_PO_4_ x 2 H_2_O | 0.1305 |
|  | Na_2_SO_4_ | 0.0705 |
|  | K_2_HPO_4_ | 0.419 |
|  | (NH_4_)_2_-citrate | 0.022 |

**Table S2:** Trace Element solution, 100-fold concentrated.

| **100 x Trace elements** | Component | Concentration [mM] |
| --- | --- | --- |
|  | EDTA-2Na salt | 13.4 |
|  | FeCl_3_ x 6 H_2_O | 3.1 |
|  | ZnSO_4_ x 7 H_2_O | 0.62 |
|  | CuCl_2_ x 2 H_2_O | 0.076 |
|  | CoCl_2_ x 6 H_2_O | 0.042 |
|  | H_3_BO_3_ | 0.142 |
|  | MnCl_2_ x 4 H_2_O | 0.0081 |

**Sequence of pLigTrans:**

TGGCGAATGGGACGCGCCCTGTAGCGGCGCATTAAGCGCGGCGGGTGTGGTGGTTACGCGCAGCGTGACCGCTACACTTGCCAGCGCCCTAGCGCCCGCTCCTTTCGCTTTCTTCCCTTCCTTTCTCGCCACGTTCGCCGGCTTTCCCCGTCAAGCTCTAAATCGGGGGCTCCCTTTAGGGTTCCGATTTAGTGCTTTACGGCACCTCGACCCCAAAAAACTTGATTAGGGTGATGGTTCACGTAGTGGGCCATCGCCCTGATAGACGGTTTTTCGCCCTTTGACGTTGGAGTCCACGTTCTTTAATAGTGGACTCTTGTTCCAAACTGGAACAACACTCAACCCTATCTCGGTCTATTCTTTTGATTTATAAGGGATTTTGCCGATTTCGGCCTATTGGTTAAAAAATGAGCTGATTTAACAAAAATTTAACGCGAATTTTAACAAAATATTAACGTTTACAATTTCAGGTGGCACTTTTCGGGGAAATGTGCGCGGAACCCCTATTTGTTTATTTTTCTAAATACATTCAAATATGTATCCGCTCATGAATTAATTCTTAGAAAAACTCATCGAGCATCAAATGAAACTGCAATTTATTCATATCAGGATTATCAATACCATATTTTTGAAAAAGCCGTTTCTGTAATGAAGGAGAAAACTCACCGAGGCAGTTCCATAGGATGGCAAGATCCTGGTATCGGTCTGCGATTCCGACTCGTCCAACATCAATACAACCTATTAATTTCCCCTCGTCAAAAATAAGGTTATCAAGTGAGAAATCACCATGAGTGACGACTGAATCCGGTGAGAATGGCAAAAGTTTATGCATTTCTTTCCAGACTTGTTCAACAGGCCAGCCATTACGCTCGTCATCAAAATCACTCGCATCAACCAAACCGTTATTCATTCGTGATTGCGCCTGAGCGAGACGAAATACGCGATCGCTGTTAAAAGGACAATTACAAACAGGAATCGAATGCAACCGGCGCAGGAACACTGCCAGCGCATCAACAATATTTTCACCTGAATCAGGATATTCTTCTAATACCTGGAATGCTGTTTTCCCGGGGATCGCAGTGGTGAGTAACCATGCATCATCAGGAGTACGGATAAAATGCTTGATGGTCGGAAGAGGCATAAATTCCGTCAGCCAGTTTAGTCTGACCATCTCATCTGTAACATCATTGGCAACGCTACCTTTGCCATGTTTCAGAAACAACTCTGGCGCATCGGGCTTCCCATACAATCGATAGATTGTCGCACCTGATTGCCCGACATTATCGCGAGCCCATTTATACCCATATAAATCAGCATCCATGTTGGAATTTAATCGCGGCCTAGAGCAAGACGTTTCCCGTTGAATATGGCTCATAACACCCCTTGTATTACTGTTTATGTAAGCAGACAGTTTTATTGTTCATGACCAAAATCCCTTAACGTGAGTTTTCGTTCCACTGAGCGTCAGACCCCGTAGAAAAGATCAAAGGATCTTCTTGAGATCCTTTTTTTCTGCGCGTAATCTGCTGCTTGCAAACAAAAAAACCACCGCTACCAGCGGTGGTTTGTTTGCCGGATCAAGAGCTACCAACTCTTTTTCCGAAGGTAACTGGCTTCAGCAGAGCGCAGATACCAAATACTGTCCTTCTAGTGTAGCCGTAGTTAGGCCACCACTTCAAGAACTCTGTAGCACCGCCTACATACCTCGCTCTGCTAATCCTGTTACCAGTGGCTGCTGCCAGTGGCGATAAGTCGTGTCTTACCGGGTTGGACTCAAGACGATAGTTACCGGATAAGGCGCAGCGGTCGGGCTGAACGGGGGGTTCGTGCACACAGCCCAGCTTGGAGCGAACGACCTACACCGAACTGAGATACCTACAGCGTGAGCTATGAGAAAGCGCCACGCTTCCCGAAGGGAGAAAGGCGGACAGGTATCCGGTAAGCGGCAGGGTCGGAACAGGAGAGCGCACGAGGGAGCTTCCAGGGGGAAACGCCTGGTATCTTTATAGTCCTGTCGGGTTTCGCCACCTCTGACTTGAGCGTCGATTTTTGTGATGCTCGTCAGGGGGGCGGAGCCTATGGAAAAACGCCAGCAACGCGGCCTTTTTACGGTTCCTGGCCTTTTGCTGGCCTTTTGCTCACATGTTCTTTCCTGCGTTATCCCCTGATTCTGTGGATAACCGTATTACCGCCTTTGAGTGAGCTGATACCGCTCGCCGCAGCCGAACGACCGAGCGCAGCGAGTCAGTGAGCGAGGAAGCGGAAGAGCGCCTGATGCGGTATTTTCTCCTTACGCATCTGTGCGGTATTTCACACCGCATATATGGTGCACTCTCAGTACAATCTGCTCTGATGCCGCATAGTTAAGCCAGTATACACTCCGCTATCGCTACGTGACTGGGTCATGGCTGCGCCCCGACACCCGCCAACACCCGCTGACGCGCCCTGACGGGCTTGTCTGCTCCCGGCATCCGCTTACAGACAAGCTGTGACCGTCTCCGGGAGCTGCATGTGTCAGAGGTTTTCACCGTCATCACCGAAACGCGCGAGGCAGCTGCGGTAAAGCTCATCAGCGTGGTCGTGAAGCGATTCACAGATGTCTGCCTGTTCATCCGCGTCCAGCTCGTTGAGTTTCTCCAGAAGCGTTAATGTCTGGCTTCTGATAAAGCGGGCCATGTTAAGGGCGGTTTTTTCCTGTTTGGTCACTGATGCCTCCGTGTAAGGGGGATTTCTGTTCATGGGGGTAATGATACCGATGAAACGAGAGAGGATGCTCACGATACGGGTTACTGATGATGAACATGCCCGGTTACTGGAACGTTGTGAGGGTAAACAACTGGCGGTATGGATGCGGCGGGACCAGAGAAAAATCACTCAGGGTCAATGCCAGCGCTTCGTTAATACAGATGTAGGTGTTCCACAGGGTAGCCAGCAGCATCCTGCGATGCAGATCCGGAACATAATGGTGCAGGGCGCTGACTTCCGCGTTTCCAGACTTTACGAAACACGGAAACCGAAGACCATTCATGTTGTTGCTCAGGTCGCAGACGTTTTGCAGCAGCAGTCGCTTCACGTTCGCTCGCGTATCGGTGATTCATTCTGCTAACCAGTAAGGCAACCCCGCCAGCCTAGCCGGGTCCTCAACGACAGGAGCACGATCATGCGCACCCGTGGGGCCGCCATGCCGGCGATAATGGCCTGCTTCTCGCCGAAACGTTTGGTGGCGGGACCAGTGACGAAGGCTTGAGCGAGGGCGTGCAAGATTCCGAATACCGCAAGCGACAGGCCGATCATCGTCGCGCTCCAGCGAAAGCGGTCCTCGCCGAAAATGACCCAGAGCGCTGCCGGCACCTGTCCTACGAGTTGCATGATAAAGAAGACAGTCATAAGTGCGGCGACGATAGTCATGCCCCGCGCCCACCGGAAGGAGCTGACTGGGTTGAAGGCTCTCAAGGGCATCGGTCGAGATCCCGGTGCCTAATGAGTGAGCTAACTTACATTAATTGCGTTGCGCTCACTGCCCGCTTTCCAGTCGGGAAACCTGTCGTGCCAGCTGCATTAATGAATCGGCCAACGCGCGGGGAGAGGCGGTTTGCGTATTGGGCGCCAGGGTGGTTTTTCTTTTCACCAGTGAGACGGGCAACAGCTGATTGCCCTTCACCGCCTGGCCCTGAGAGAGTTGCAGCAAGCGGTCCACGCTGGTTTGCCCCAGCAGGCGAAAATCCTGTTTGATGGTGGTTAACGGCGGGATATAACATGAGCTGTCTTCGGTATCGTCGTATCCCACTACCGAGATATCCGCACCAACGCGCAGCCCGGACTCGGTAATGGCGCGCATTGCGCCCAGCGCCATCTGATCGTTGGCAACCAGCATCGCAGTGGGAACGATGCCCTCATTCAGCATTTGCATGGTTTGTTGAAAACCGGACATGGCACTCCAGTCGCCTTCCCGTTCCGCTATCGGCTGAATTTGATTGCGAGTGAGATATTTATGCCAGCCAGCCAGACGCAGACGCGCCGAGACAGAACTTAATGGGCCCGCTAACAGCGCGATTTGCTGGTGACCCAATGCGACCAGATGCTCCACGCCCAGTCGCGTACCGTCTTCATGGGAGAAAATAATACTGTTGATGGGTGTCTGGTCAGAGACATCAAGAAATAACGCCGGAACATTAGTGCAGGCAGCTTCCACAGCAATGGCATCCTGGTCATCCAGCGGATAGTTAATGATCAGCCCACTGACGCGTTGCGCGAGAAGATTGTGCACCGCCGCTTTACAGGCTTCGACGCCGCTTCGTTCTACCATCGACACCACCACGCTGGCACCCAGTTGATCGGCGCGAGATTTAATCGCCGCGACAATTTGCGACGGCGCGTGCAGGGCCAGACTGGAGGTGGCAACGCCAATCAGCAACGACTGTTTGCCCGCCAGTTGTTGTGCCACGCGGTTGGGAATGTAATTCAGCTCCGCCATCGCCGCTTCCACTTTTTCCCGCGTTTTCGCAGAAACGTGGCTGGCCTGGTTCACCACGCGGGAAACGGTCTGATAAGAGACACCGGCATACTCTGCGACATCGTATAACGTTACTGGTTTCACATTCACCACCCTGAATTGACTCTCTTCCGGGCGCTATCATGCCATACCGCGAAAGGTTTTGCGCCATTCGATGGTGTCCGGGATCTCGACGCTCTCCCTTATGCGACTCCTGCATTAGGAAGCAGCCCAGTAGTAGGTTGAGGCCGTTGAGCACCGCCGCCGCAAGGAATGGTGCATGCAAGGAGATGGCGCCCAACAGTCCCCCGGCCACGGGGCCTGCCACCATACCCACGCCGAAACAAGCGCTCATGAGCCCGAAGTGGCGAGCCCGATCTTCCCCATCGGTGATGTCGGCGATATAGGCGCCAGCAACCGCACCTGTGGCGCCGGTGATGCCGGCCACGATGCGTCCGGCGTAGAGGATCGAGATCTCGATCCCGCGAAATTAATACGACTCACTATAGGGGAATTGTGAGCGGATAACAATTCCCCTCTAGAAATAATTTTGTTTAACTTTAAGAAGGAGATATACCATGACCCACGAAGTGAAAATCGAAGAAGGTAAACTGGTAATCTGGATTAACGGCGATAAAGGCTATAACGGTCTCGCTGAAGTCGGTAAGAAATTCGAGAAAGATACCGGAATTAAAGTCACCGTTGAGCATCCGGATAAACTGGAAGAGAAATTCCCACAGGTTGCGGCAACTGGCGATGGCCCTGACATTATCTTCTGGGCACACGACCGCTTTGGTGGCTACGCTCAATCTGGCCTGTTGGCTGAAATCACCCCGGACAAAGCGTTCCAGGACAAGCTGTATCCGTTTACCTGGGATGCCGTACGTTACAACGGCAAGCTGATTGCTTACCCGATCGCTGTTGAAGCGTTATCGCTGATTTATAACAAAGATCTGCTGCCGAACCCGCCAAAAACCTGGGAAGAGATCCCGGCGCTGGATAAAGAACTGAAAGCGAAAGGTAAGAGCGCGCTGATGTTCAACCTGCAAGAACCGTACTTCACCTGGCCGCTGATTGCTGCTGACGGGGGTTATGCGTTCAAGTATGAAAACGGCAAGTACGACATTAAAGACGTGGGCGTGGATAACGCTGGCGCGAAAGCGGGTCTGACCTTCCTGGTTGACCTGATTAAAAACAAACACATGAATGCAGACACCGATTACTCCATCGCAGAAGCTGCCTTTAATAAAGGCGAAACAGCGATGACCATCAACGGCCCGTGGGCATGGTCCAACATCGACACCAGCAAAGTGAATTATGGTGTAACGGTACTGCCGACCTTCAAGGGTCAACCATCCAAACCGTTCGTTGGCGTGCTGAGCGCAGGTATTAACGCCGCCAGTCCGAACAAAGAGCTGGCAAAAGAGTTCCTCGAAAACTATCTGCTGACTGATGAAGGTCTGGAAGCGGTTAATAAAGACAAACCGCTGGGTGCCGTAGCGCTGAAGTCTTACGAGGAAGAGTTGGCGAAAGATCCACGTATTGCCGCCACTATGGAAAACGCCCAGAAAGGTGAAATCATGCCGAACATCCCGCAGATGTCCGCTTTCTGGTATGCCGTGCGTACTGCGGTGATCAACGCCGCCAGCGGTCGTCAGACTGTCGATGAAGCCCTGAAAGACGCGCAGACTAATTCGAGCTCGCATCACCATCACCATCACCATTCCCATCAAACAAGTTTGTACAAAAAAGCAGGCTCCGCGGCCGCCTTGTTTAACTTTAAGAAGGAGCCCTTCACCATGACGACACAAGATGTGATAGTCAATGATCAGAATGATCAGAAACAGTGTAGTAATGACGTCATTTTCCGATCGAGATTGCCTGATATATACATCCCTAACCACCTCCCACTCCACGACTACATCTTCGAAAATATCTCAGAGTTCGCCGCTAAGCCATGCTTGATCAACGGTCCCACCGGCGAAGTATACACCTACGCCGATGTCCACGTAACATCTCGGAAACTCGCCGCCGGTCTTCATAACCTCGGCGTGAAGCAACACGACGTTGTAATGATCCTCCTCCCGAACTCTCCTGAAGTAGTCCTCACTTTCCTTGCCGCCTCCTTCATCGGCGCAATCACCACCTCCGCGAACCCGTTCTTCACTCCGGCGGAGATTTCTAAACAAGCCAAAGCCTCCGCGGCGAAACTCATCGTCACTCAATCCCGTTACGTCGATAAAATCAAGAACCTCCAAAACGACGGCGTTTTGATCGTCACCACCGACTCCGACGCCATCCCCGAAAACTGCCTCCGTTTCTCCGAGTTAACTCAGTCCGAAGAACCACGAGTGGACTCAATACCGGAGAAGATTTCGCCAGAAGACGTCGTGGCGCTTCCTTTCTCATCCGGCACGACGGGTCTCCCCAAAGGAGTGATGCTAACACACAAAGGTCTAGTCACGAGCGTGGCGCAGCAAGTCGACGGCGAGAATCCGAATCTTTACTTCAACAGAGACGACGTGATCCTCTGTGTCTTGCCTATGTTCCATATATACGCTCTCAACTCCATCATGCTCTGTAGTCTCAGAGTTGGTGCCACGATCTTGATAATGCCTAAGTTCGAAATCACTCTCTTGTTAGAGCAGATACAAAGGTGTAAAGTCACGGTGGCTATGGTCGTGCCACCGATCGTTTTAGCTATCGCGAAGTCGCCGGAGACGGAGAAGTATGATCTGAGCTCGGTTAGGATGGTTAAGTCTGGAGCAGCTCCTCTTGGTAAGGAGCTTGAAGATGCTATTAGTGCTAAGTTTCCTAACGCCAAGCTTGGTCAGGGCTATGGGATGACAGAAGCAGGTCCGGTGCTAGCAATGTCGTTAGGGTTTGCTAAAGAGCCGTTTCCAGTGAAGTCAGGAGCATGTGGTACGGTGGTGAGGAACGCCGAGATGAAGATACTTGATCCAGACACAGGAGATTCTTTGCCTAGGAACAAACCCGGCGAAATATGCATCCGTGGCAACCAAATCATGAAAGGCTATCTCAATGACCCCTTGGCCACGGCATCGACGATCGATAAAGATGGTTGGCTTCACACTGGAGACGTCGGATTTATCGATGATGACGACGAGCTTTTCATTGTGGATAGATTGAAAGAACTCATCAAGTACAAAGGATTTCAAGTGGCTCCAGCTGAGCTAGAGTCTCTCCTCATAGGTCATCCAGAAATCAATGATGTTGCTGTCGTCGCCATGAAGGAAGAAGATGCTGGTGAGGTTCCTGTTGCGTTTGTGGTGAGATCGAAAGATTCAAATATATCCGAAGATGAAATCAAGCAATTCGTGTCAAAACAGGTTGTGTTTTATAAGAGAATCAACAAAGTGTTCTTCACTGACTCTATTCCTAAAGCTCCATCAGGGAAGATATTGAGGAAGGATCTAAGAGCAAGACTAGCAAATGGATTAATGAACTGAGAAGGAGATATACCATGGCTCCTGCTCCTCAACTTCCTACACCATCTGAAACAATAACCACCGATGCATCATCGGAAAACAACAATGTTACCATCACTGAAAAGATATACACACGAGTCCGTCTGGCTACGAAATCTGATCTGTCTCATATATACCAATTGTTTTATCAAATCCATGAATACCATAACTATACTCATTTATACAAAGCTACTGAGTCCTCCTTAGCCAACTTGCTCTTTAAAGAAAACCCTCTTCCCCTTTTCTACGGGCCATCCGTACTTCTACTTGAAGTCTCTCCAACCCCTTTTAACGAACCCAAAAATACCACAAACGAAGGGTTCAAGCCTGTCCTTACAACTTTTGACCTTAAATTCCCTGTGGTGGAAGGACAAGTTGAGGAGTTCAGGTCCAAATATGACGATAAGAATGATGCTTACATTGCAGGATATGCTTTCTTTTACGCTAATTATTCATGTTTCTATGACAAGCCAGGATTCTATTTTGAGAGTCTTTACTTCAGAGAGAGTTATAGAAAGTTGGGAATGGGGAGTTTGTTGTTTGGAACAGTTGCATCTATTGCTGCAAACAATGGCTTCGTATCGGTAGAGGGAATAGTAGCAGTTTGGAATAAAAAGTCATATGATTTTTACATAAATATGGGAGTTGAAATTTTTGATGAGTTTAGGTATGGCAAGTTGCATGGTGAAAATCTTCAAAAGTATGCTGATAAAAAGGACGAAAACGGCGAAGGGAGCTGTTAGCTCGAGCACCACCACCACCACCACTGAGATCCGGCTGCTAACAAAGCCCGAAAGGAAGCTGAGTTGGCTGCTGCCACCGCTGAGCAATAACTAGCATAACCCCTTGGGGCCTCTAAACGGGTCTTGAGGGGTTTTTTGCTGAAAGGAGGAACTATATCCGGAT

**A representative vessel script for automated process control by the Dasgip system.**

```
Dim StartDelay_h As Double = 0 'delay after inoculation [h]
Dim lowLP_PV As Double = 40 'DOPV < lowDOTrg start waiting LP_DO peak Trigger
Dim peakDLP_PV As Double = 4 'DeltaDOPV %/min > peakDOTrg DO peak Trigger
Dim µmax As Double = 0.30 ' growth rate
Dim Feed_start As Double = 0.72 'ml/h, V_start*c_Glc_Stat / feed (500g/l)
Dim tPhase_h As Double
'internal variables
Dim Feed As Double = 0
dim DOPV as double
Dim LP_PV as Double 'low Pass PV
Dim DLP_PV as Double '[%DO/min]
Dim SubLP_PV as Double '[%DO/min]
Dim xk as Double
Dim sz as Double
Dim c1 as Double
Dim c2 as Double
Dim yk as Double
Dim yk_1 as Double
Dim tk as Double
Dim tk_1 as Double
Dim SubLP_PV_1 as Double
Dim Ti as Double = 1/60 '[h] low pass filter

'Storege
dim a(4) As Double

if P isNot Nothing then
  with P

  DOPV = p.DOPV
  tPhase_h = p.RunTime_h - p.PhaseStart_h

 'Init Storege
      if s is Nothing then
      'a(1) tk_1  t (k-1)
      'a(2) yk_1  LowPass PV (k-1)
      'a(3) SubLP_PV_1
      a(1) = 0
      a(2) = 0
      a(3) = 0
      s = a
    end if

      'read storage
          tk_1 = s(1)
          yk_1 = s(2)
    SubLP_PV_1 = s(3)

    'low Pass
    tk = p.Runtime_h
    if tk_1 <> 0 then
      xk       = DOPV
      sz       = tk - tk_1
      c1       = sz / (Ti + sz)
      c2       = Ti / (Ti + sz)
      yk       = c1 * xk + c2 * yk_1
      LP_PV    = yk
      SubLP_PV = (yk - yk_1) / (sz*60)
      DLP_PV   = (c1 * SubLP_PV) + (c2 * SubLP_PV_1)
    else 'init low Pass
      yk = DOPV
      LP_PV = DOPV
      DLP_PV = 0
    SubLP_PV = 0
    end if

      'Write Storage
    a(1) = tk
    a(2) = yk
    a(3) = DLP_PV
    s = a

    'example value outpot to internal values
    p.intC = LP_PV
    p.IntD = DLP_PV

    select case p.phase
    case 0
      p.phase = p.phase + 1
      p.LogMessage("Entering phase " & p.phase & ": Waiting for InoculationTime > " & format(StartDelay_h, "#.##") & "[h]")
    case 1 'batch phase
      if p.InoculationTime_H > StartDelay_h then
        p.phase = p.phase + 1
        p.LogMessage("Entering batch phase " & p.phase & ": Waiting for DOPV <" & lowLP_PV)
      end if
    case 2 'batch phase: activate DO peak trigger
      if LP_PV < lowLP_PV then
        p.phase = p.phase + 1
        p.LogMessage("Entering batch phase II " & p.phase & ": Waiting for DLP_PV > " & peakDLP_PV)
      end if
    case 3 'DO peak trigger
      if DLP_PV > peakDLP_PV then
        p.phase = p.phase + 1
        p.LogMessage("Entering exp. phase " & p.phase & ": start µ_max based exponential feeding")
      end if
    case 4 'phase of exponential feeding
    tPhase_h = p.RunTime_h - p.PhaseStart_h
       p.FCSP = Feed_start * exp(µmax * tPhase_H)
    if p.FCSP > 14.0 then
    p.phase = p.phase + 1
        p.LogMessage("Entering induction phase " & p.phase & ": start continous feeding")
      end if
    case 5 ' continous feeding
    p.FCSP = 14.00
   if p.TSP < 24 then
      p.phase = p.phase + 1
      p.LogMessage("Entering cold expression " & p.phase & ":for over night " )
      end if
   case 6 ' cold induction
    p.FCSP = 2.4
    p.FASP = 0
   if p.TSP > 20 then
      p.phase = p.phase + 1
      p.LogMessage("Entering Production phase" & p.phase & ":Substrate feed" )
      end if
   case 7 ' Reaction
    p.FCSP = 6.7
    if tPhase_H > 60/60 and tPhase_H < 2160/60 then
    p.FASP = (p.VPV*0.0125) ' 0.400 M FerA and TrN 5 mM/h
    else p.FASP = 0
   end if

end select

end with
end if
```
